# Low-molecular-weight heparin use in coronavirus disease 2019 is associated with curtailed viral persistence: a retrospective multicentre observational study

**DOI:** 10.1093/cvr/cvab308

**Published:** 2021-10-05

**Authors:** David Pereyra, Stefan Heber, Waltraud C Schrottmaier, Jonas Santol, Anita Pirabe, Anna Schmuckenschlager, Kerstin Kammerer, Daphni Ammon, Thomas Sorz, Fabian Fritsch, Hubert Hayden, Erich Pawelka, Philipp Krüger, Benedikt Rumpf, Marianna T Traugott, Pia Glaser, Christa Firbas, Christian Schörgenhofer, Tamara Seitz, Mario Karolyi, Ingrid Pabinger, Christine Brostjan, Patrick Starlinger, Günter Weiss, Rosa Bellmann-Weiler, Helmut J F Salzer, Bernd Jilma, Alexander Zoufaly, Alice Assinger

**Affiliations:** 1 Department of Vascular Biology and Thrombosis Research, Center of Physiology and Pharmacology, Medical University of Vienna, Schwarzspanierstraße 17, 1090 Vienna, Austria; 2 Division of Visceral Surgery, Department of Surgery, Medical University of Vienna, General Hospital Vienna, Vienna, Austria; 3 Institute of Physiology, Centre of Physiology and Pharmacology, Medical University of Vienna, Vienna, Austria; 4 Division of Vascular Surgery, Department of Surgery, Medical University of Vienna, General Hospital Vienna, Vienna, Austria; 5 Department of Medicine IV, Kaiser Franz Josef Hospital, Vienna, Austria; 6 Department of Medicine I, Medical University of Vienna, General Hospital Vienna, Vienna, Austria; 7 Department of Clinical Pharmacology, Medical University of Vienna, General Hospital Vienna, Vienna, Austria; 8 Department of Internal Medicine II, Medical University of Innsbruck, Innsbruck, Austria; 9 Department of Pulmonology, Kepler University Hospital, Johannes Kepler University, Linz, Austria

**Keywords:** COVID-19-associated coagulopathy, Anticoagulation, Low-molecular-weight heparin, Thromboinflammation, SARS-CoV-2 viral persistence

## Abstract

**Aims:**

Anticoagulation was associated with improved survival of hospitalized coronavirus disease 2019 (COVID-19) patients in large-scale studies. Yet, the development of COVID-19-associated coagulopathy (CAC) and the mechanism responsible for improved survival of anticoagulated patients with COVID-19 remain largely elusive. This investigation aimed to explore the effects of anticoagulation and low-molecular-weight heparin (LMWH) in particular on patient outcome, CAC development, thromboinflammation, cell death, and viral persistence.

**Methods and results:**

Data of 586 hospitalized COVID-19 patients from three different regions of Austria were evaluated retrospectively. Of these, 419 (71.5%) patients received LMWH and 62 (10.5%) received non-vitamin-K oral anticoagulants (NOACs) during hospitalization. Plasma was collected at different time points in a subset of 106 patients in order to evaluate markers of thromboinflammation (H3Cit-DNA) and the cell death marker cell-free DNA (cfDNA). Use of LMWH was associated with improved survival upon multivariable Cox regression (hazard ratio = 0.561, 95% confidence interval: 0.348–0.906). Interestingly, neither LMWH nor NOAC was associated with attenuation of D-dimer increase over time, or thromboinflammation. In contrast, anticoagulation was associated with a decrease in cfDNA during hospitalization, and curtailed viral persistence was observed in patients using LMWH leading to a 4-day reduction of virus positivity upon quantitative polymerase chain reaction [13 (interquartile range: 6–24) vs. 9 (interquartile range: 5–16) days, *P* = 0.009].

**Conclusion:**

Time courses of haemostatic and thromboinflammatory biomarkers were similar in patients with and without LMWH, indicating either no effects of LMWH on haemostasis or that LMWH reduced hypercoagulability to levels of patients without LMWH. Nonetheless, anticoagulation with LMWH was associated with reduced mortality, improved markers of cell death, and curtailed viral persistence, indicating potential beneficial effects of LMWH beyond haemostasis, which encourages use of LMWH in COVID-19 patients without contraindications.

## Graphical Abstract

**Figure:**
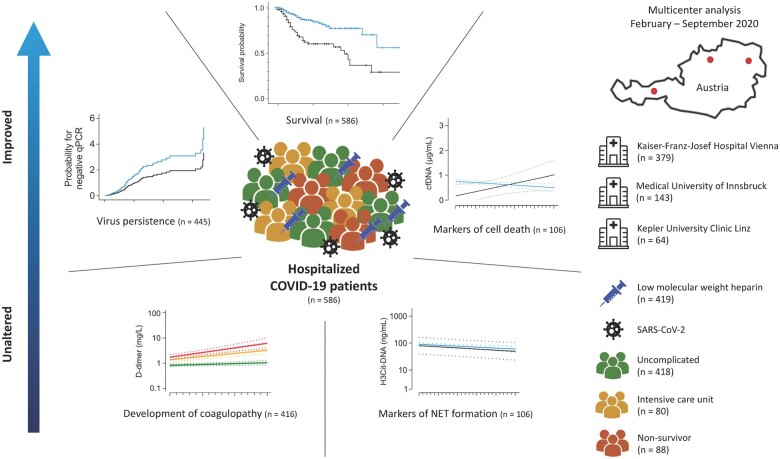

## 1. Introduction

Coronavirus disease 2019 (COVID-19) caused by severe acute respiratory syndrome coronavirus 2 (SARS-CoV-2) has evoked an international pandemic of unforeseen global impact. While the rapid development of effective vaccines provides hope for the management of the pandemic, availability as well as the mutational burden require further research on patient care in COVID-19.^1^ In particular, the hypercoagulatory state induced by SARS-CoV-2 and associated thrombotic complications seen in COVID-19 represent crucial complications in hospitalized patients impacting disease course and survival.^2^^,^^3^ COVID-19-associated coagulopathy (CAC), characterized by a slight reduction in platelet counts accompanied by increased levels of D-dimer and a prolongation in prothrombin time (PT), has emerged as a novel type of coagulopathy distinctly different from disseminated intravascular and sepsis-induced coagulopathy.^4^ Hence, specific anticoagulation in COVID-19 is intensively discussed,^5^ and retrospective analyses revealed that prophylactic and therapeutic anticoagulations are associated with improved survival.^6^^,^^7^ Intriguingly, use of low-molecular-weight heparin (LMWH) in COVID-19 showed no impact of this drug on *in vivo* activation of coagulation or fibrinolysis.^8^^,^^9^ Thus, clinical evidence for the mechanism of action of anticoagulants and improved survival in COVID-19 is still missing.

Neutrophil extracellular traps (NETs), caused by decondensation of nuclear DNA from neutrophils, are associated with disease progression in COVID-19.^10^ NET formation is strongly linked to the presence of thromboinflammation and represents an interface between hyperinflammation and hypercoagulatory loop seen in COVID-19. Thereby, NET formation was hypothesized to be a crucial feature of CAC.

Within this observational study, we report on a multicentre cohort of hospitalized COVID-19 patients in Austria. We evaluated the predictive potential of previously reported haemostatic biomarkers for mortality and investigated markers of thromboinflammation and cell death in these patients. Further, we explored the relationship between LMWH intake and potential underlying mechanisms responsible for the observed improved outcome of COVID-19 patients receiving anticoagulation by analysing their impact on thromboinflammation, cell death, and duration of viral persistence.

## 2. Methods

### 2.1 Patients

In this retrospective multicentre study, clinical data from COVID-19 patients (*n* = 586) admitted to three different large-scale treatment centres in Austria (Kaiser Franz Josef Hospital, Vienna, Medical University of Innsbruck, Kepler University Hospital, Linz) between 14 February 2020 and 18 September 2020 were analysed. Patient demographics including comorbidities, use of anticoagulation, and additional medication relevant for COVID-19 treatment were recorded. Routine laboratory analysis was performed every second day. Nasopharyngeal swabs and quantitative polymerase chain reaction (qPCR) for SARS-CoV-2 were performed according to the Charité protocol^11^ at multiple time points (every other day excluding weekends) in order to evaluate viral persistence. Of note, no patient was using heparin-containing nasal sprays or similar compounds that might affect qPCR. In a subset of 106 patients hospitalized at Kaiser Franz Josef Hospital, Vienna, additional blood withdrawals and plasma preparation for evaluation of pathophysiological markers in circulation [i.e., citrullinated histone 3 DNA complexes (H3Cit-DNA), cell-free DNA (cfDNA)] were performed at baseline and every second day or third day in case of a weekend in between for up to four blood withdrawals (for more detail, refer to Supplementary material online, *Methods* and Supplementary material online, *Table S1*). Disease severity according to the World Health Organization (WHO) classification was assessed at admission (www.who.int; Clinical management of COVID-19 Interim guidance 27 May 2020). Accordingly, patients were classified as mild, moderate, severe, or critical based on clinical evaluation and disease dynamic within the first week after admission to hospital. Patients without COVID-19 symptoms at admission were added to the cohort of mild patients. Outcome data were available for all patients at the time of analysis. The study was conducted in accordance with the Declaration of Helsinki. The recovery of data at Kaiser Franz Josef Hospital in Vienna was part of the randomized controlled ACOVACT study (ClinicalTrials.gov: NCT04351724) approved by the local ethics committee (EK1315/2020), which aims to compare the effect of different antiviral agents and adjunctive treatments on outcome of hospitalized COVID-19 patients. Data gathered at the remaining two centres were prospectively evaluated and retrospectively analysed in this investigation. Accordingly, the study was approved by the ethics committee of the Medical University of Innsbruck (EK1167/2020) and the Kepler University Hospital Linz (EK1085/2020). Patients in the ACOVACT study gave written informed consent prior to study inclusion, while patient consent was waved for all remaining participants.

### 2.2 Statistical analysis

A detailed description of statistical analyses is found in the Supplementary material online. Briefly, patient demographics and laboratory parameters on admission were compared between survivors and non-survivors using Mann–Whitney *U* test, *χ*^2^, or Fisher’s exact test for metric or nominal and ordinal variables, respectively. Missing data were evaluated and approached using multiple imputations, as described in the Supplementary material online, *Methods* section.

The binary outcome death or survival was predicted using univariable and multivariable logistic regression models. Comparison of prognostic values was undertaken using receiver operating characteristics analyses. The time course of D-dimer levels during hospitalization was explored using a mixed model approach, allowing estimation of differences in D-dimer dynamic between evaluated groups. Similarly, the mixed model approach was fit for markers of NET formation and cell death. For a more detailed description of the mixed model approach, refer to the Supplementary material online, *Methods* section. For survival analyses, multivariable Cox proportional hazard regression models were applied. Likewise, estimation of viral persistence over time and comparison between two groups was achieved using multivariable Cox regression. It has to be mentioned that unbalanced distribution of some confounders might render statistical models unstable. Thus, the results have to be interpreted with prudence, and observed effects need to be validated in interventional trials.

All statistical analyses were performed with IBM SPSS statistics 26, and graphs were generated with GraphPad Prism 8.4. Due to the exploratory character of all analyses, no adjustment for multiple testing was performed, and results have to be interpreted accordingly. Only two-sided tests were used, and *P*-values ≤0.05 were considered statistically significant.

## 3. Results

### 3.1 Patient demographics

**Figure 1:**
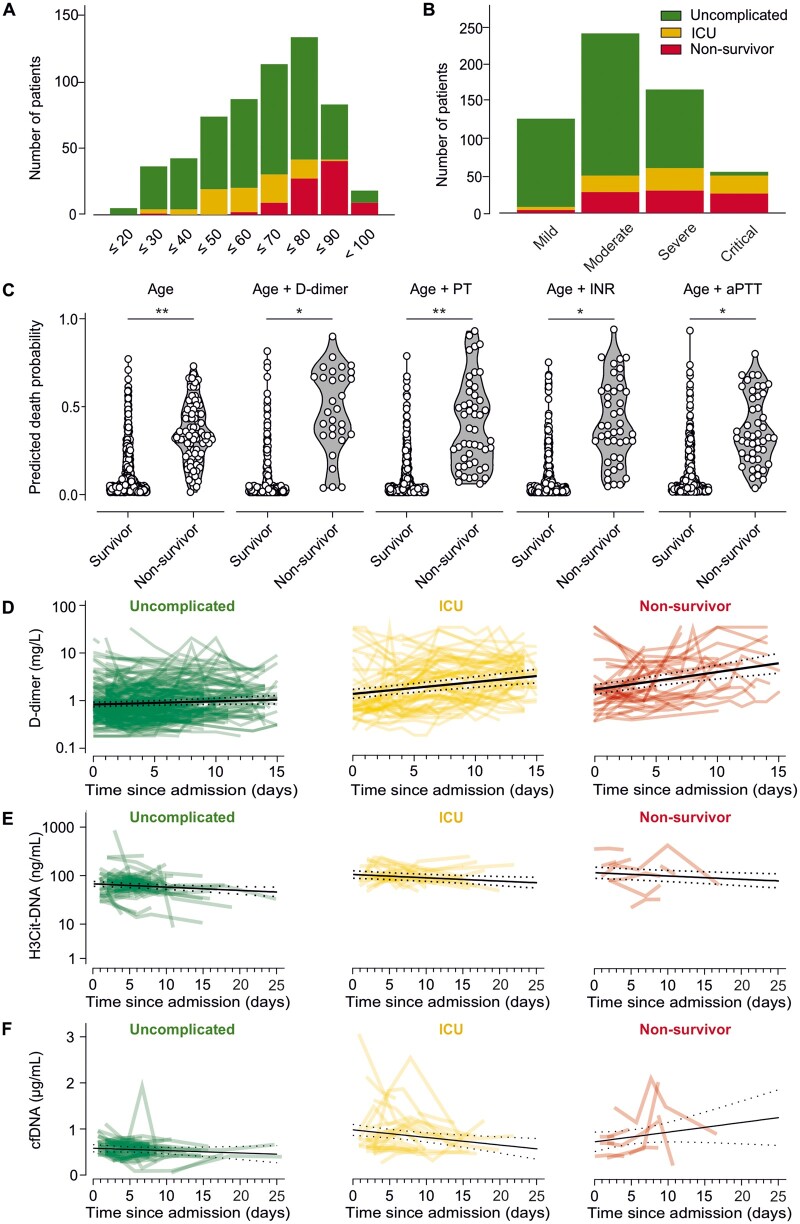
Prognostic value of haematological parameters in COVID-19 patients at admission and development of CAC and thromboinflammation. (*A*) Total number of patients per age group and incidence of uncomplicated hospitalization, requirement of ICU, and mortality. (*B*) Total number of patients and incidence of uncomplicated hospitalization, requirement of ICU, and mortality according to COVID-19 classification at admission. (*C*) Death probability for each patient estimated by logistic regression with the predictors age, age + D-dimer, age + prothrombin time (PT), age + INR, age + aPTT. Prognostic potential was compared using receiver operating characteristic curve analyses. (*D–F*) Time courses of D-dimer (*D*), citrullinated histone 3 (H3Cit)–DNA complexes (*E*), and cell-free DNA (cfDNA) (*F*) are shown for patients with uncomplicated course of disease (green), those who were admitted to an ICU (yellow), and those who died (red). Time courses were modelled using mixed linear models, leading to respective *P*-values for comparison of dynamics over time between outcome cohorts. Lines with dotted error bands represent least-squared means of log-transformed D-dimer values with 95% confidence intervals (*N* = 586 in *A–D, N* = 106 in *E and F*, *P < 0.05, **P < 0.001).

In total, 586 consecutive patients were retrospectively analysed in this study (Kaiser Franz Josef Hospital, Vienna: *n* = 379, Medical University of Innsbruck: *n* = 143, Johannes Kepler University Hospital Linz: *n* = 64). Patient demographics are given in*Tables 1 and**2*, and cohort comparison can be found in Supplementary material online, *Table S2*. Median age at admission was 64 years [interquartile range (IQR) 49–77], and 60.2% of patients were male. Incidence of complicated hospitalization and fatal outcome increased with higher age (*Figure 1A*). Disease severity at admission was indicative for outcome (*Figure 1B*). Intensive care unit (ICU) treatment was required in 111 patients (18.9%) and 66 patients (11.3%) required invasive ventilation. Overall, 88 patients (15.0%) died during their hospital stay. The main cause of death in this cohort was respiratory failure in 49 patients (55.7%), followed by multiorgan failure (18 patients, 20.5%), cardiac decompensation (8 patients, 9.1%), sepsis (7 patients, 8.0%), and thromboembolic complications (3 patients, 3.4%). Cause of death is unknown in three patients (3.4%). Missing data are presented in Supplementary material online, *Table S3a and b*, and frequency of missing data was compared between survivors and non-survivors showing a higher proportion of missing values for vital signs in non-survivors (Supplementary material online, *Table S3a*). However, missing data for routine laboratory results were distributed equally between both groups (Supplementary material online, *Table S3b*). In order to account for missing data in the comparison of survivors and non-survivors, multiple imputation was conducted, which is a statistical state-of-the-art method to account for missing data.^12^ Respective results for each variable in 20 data sets are shown in Supplementary material online, *Figure S1*.

**Table 1:** Patient demographics

Parameter	Missing data	Entire cohort	Survivors	Non-survivors	*P*-value
		(*N* = 586)	(*N* = 498)	(*N* = 88)	
	*N*	*N* (%)	*N* (%)	*N* (%)	
		Median (IQR)	Median (IQR)	Median (IQR)	
Sex	0				0.157
Female		233 (39.8%)	204 (41.0%)	29 (33.0%)	
Male		353 (60.2%)	294 (59.0%)	59 (67.0%)	
Age (years)	0	64 (49–77)	61 (47–74)	81 (75–86)	**<0.001**
Comorbidities					
Current smoker	117	34 (5.8%)	31 (6.2%)	3 (3.4%)	0.472
Obesity (BMI > 25)	69	260 (44.4%)	233 (46.8%)	27 (30.7%)	0.383
Diabetes type II	1	119 (20.3%)	92 (18.5%)	27 (30.7%)	**0.007**
Hypertension	16	285 (48.6%)	223 (44.8%)	62 (70.5%)	**<0.001**
Cardiovascular disease (any)	23	151 (25.8%)	100 (20.1%)	51 (58.0%)	**<0.001**
Coronary heart disease	25	77 (13.1%)	53 (10.6%)	24 (27.3%)	**<0.001**
Chronic heart failure	2	37 (6.3%)	23 (4.6%)	14 (15.9%)	**<0.001**
Atrial fibrillation	1	76 (13.0%)	46 (9.2%)	30 (34.1%)	**<0.001**
Peripheral arterial disease	1	25 (4.3%)	15 (3.0%)	10 (11.4%)	0.002*
Chronic obstructive pulmonary disease	23	47 (8.0%)	34 (6.8%)	13 (14.8%)	**0.009**
Asthma	1	26 (4.4%)	23 (4.6%)	3 (3.4%)	0.783^*^
Hypo-/hyperthyroidism	1	59 (10.1%)	49 (9.8%)	10 (11.4%)	0.636
Chronic renal insufficiency	1	65 (11.1%)	37 (7.4%)	28 (31.8%)	**<0.001**
Chronic liver disease	2	29 (4.9%)	25 (5.0%)	4 (4.5%)	0.884
Malignancy	1	59 (10.1%)	42 (8.4%)	17 (19.3%)	**0.002**
Symptoms at admission					
Asymptomatic	1	39 (6.7%)	37 (7.4%)	2 (2.3%)	0.077
Fatigue	3	299 (51.0%)	253 (50.8%)	46 (52.3%)	0.658
Cough	5	384 (65.5%)	339 (68.1%)	45 (51.1%)	**0.006**
Fever	3	353 (60.2%)	294 (59.0%)	59 (67.0%)	0.098
Requirement of oxygen	12	238 (40.6%)	180 (36.1%)	58 (65.9%)	**<0.001**
Dyspnoea	9	233 (39.8%)	194 (39.0%)	39 (44.3%)	0.222
Diarrhoea	4	92 (15.7%)	82 (16.5%)	10 (11.4%)	0.250
Sore throat	5	53 (9.0%)	52 (10.4%)	1 (1.1%)	**0.005**
Nausea or vomiting	4	70 (11.9%)	61 (12.2%)	9 (10.2%)	0.629
Vital signs at admission					
Temperature (°C)	145	36.7 (36.3–37.8)	36.7 (36.3–37.8)	36.7 (36.3–37.8)	0.996
Pulse rate (beats per minute)	109	85 (75–97)	85 (75–97)	81 (71–93)	0.165
Systolic arterial pressure (mmHg)	103	130 (120–140)	130 (120–140)	130 (120–145)	0.501
Diastolic arterial pressure (mmHg)	105	80 (70–85)	80 (70–85)	80 (70–81)	0.517
Respiratory rate (b.p.m.)	256	20 (16–24)	20 (16–24)	20 (18–26)	**0.010**
SpO_2_ at ambient air (%)	113	95 (93–97)	96 (94–97)	94 (92–95)	**<0.001**
COVID-19 classification at admission^a^	0				**<0.001**
Mild		126 (21.5%)	122 (24.5%)	4 (4.5%)	
Moderate		240 (41.0%)	212 (42.6%)	28 (31.8%)	
Severe		165 (28.2%)	135 (27.1%)	30 (34.1%)	
Critical		55 (9.4%)	29 (5.8%)	26 (29.5%)	
Anticoagulation					
LMWH	4	419 (71.5%)	375 (75.6%)	44 (51.2%)	**<0.001**
NOAC	0	62 (10.6%)	53 (10.6%)	9 (10.2%)	0.907
Additional treatment					
Corticosteroids	1	165 (28.2%)	144 (29.0%)	21 (23.9%)	0.326
Remdesivir/favipiravir	0	77 (13.1%)	58 (11.6%)	19 (21.6%)	**0.011**
Lopinavir/ritonavir	0	85 (14.5%)	78 (15.7%)	7 (8.0%)	0.058
Camostat	0	37 (6.3%)	37 (7.4%)	0 (0.0%)	**0.008**
Clinical characteristics					
Total hospitalization (days)	0	10 (6–17)	10 (6–18)	9 (6–14)	0.116
Admission to ICU	0	111 (18.9%)	80 (16.1%)	31 (35.2%)	**<0.001**
Invasive ventilation	0	66 (11.3%)	44 (8.8%)	22 (25.0%)	**<0.001**

For detailed information on missing data, refer to Supplementary material online, *Table S3*. Bold values indicate statistically significant results.

BMI, body mass index; COVID-19, coronavirus disease 2019; ICU, intensive care unit; IQR, interquartile range; mmHg, millimetre mercury; SpO_2_, peripheral capillary oxygen saturation.

a COVID-19 classification was performed according to the guidelines issued by the WHO in mild (fever <38°C, no dyspnoea, no pneumonia), moderate (fever, respiratory symptoms, pneumonia), severe (respiratory distress with respiratory rate ≥30 b.p.m., SpO_2_ < 93% at rest), and critical (respiratory failure with requirement of mechanical ventilation, requirement of ICU).

* Fisher’s exact test.

**Table 2:** Laboratory parameters at admission

Parameter	Missing data	Entire cohort	Survivors	Non-survivors	*P*-value
		(*N* = 586)	(*N* = 498)	(*N* = 88)	
	*N*	Median (IQR)	Median (IQR)	Median (IQR)	
Haemoglobin (g/dL)	173	13.6	13.7	12.8	**0.002**
		(12.3–14.8)	(12.5–14.8)	(11.2–14.2)	
Red blood cell count (×10^12^/L)	139	4.54	4.57	4.21	**0.001**
		(4.09–4.96)	(4.14–4.99)	(3.63–4.77)	
Platelet count (×10^9^/L)	159	186	192	171	**0.045**
		(150–247)	(153–251)	(135–222)	
Leucocyte count (×10^9^/L)	159	5.7	5.5	6.5	**0.003**
		(4.3–7.9)	(4.2–7.7)	(4.6–10.5)	
Lymphocyte count (×10^9^/L)	144	1.15	1.21	0.87	**0.001**
		(0.74–9.25)	(0.78–11.55)	(0.65–3.31)	
C-reactive protein (mg/L)	162	50.65	48.2	84.0	**<0.001**
		(20.18–95.93)	(16.8–89.4)	(34.0–169.5)	
D-dimer (mg/L)	366	0.79	0.70	1.55	**<0.001**
		(0.52–1.56)	(0.48–1.29)	(0.89–2.04)	
Prothrombin time (%)	229	101	103	93	**<0.001**
		(88–110)	(90–111)	(68–100)	
International normalized ratio	274	1.00	1.00	1.06	**<0.001**
		(1.00–1.07)	(1.00–1.05)	(1.00–1.10)	
Activated partial thromboplastin time (s)	256	30.7	30.3	33.0	**0.012**
		(27.5–34.0)	(27.4–33.8)	(28.8–39.2)	

For detailed information on missing data, refer to Supplementary material online, *Table S3*. Bold values indicate statistically significant results.

BMI, body mass index; COVID-19, coronavirus disease 2019; ICU, intensive care unit; IQR, interquartile range; mmHg, millimetre mercury; SpO_2_, peripheral capillary oxygen saturation.

### 3.2 Predictive value of haemostatic biomarkers for mortality is limited compared to older age

Age predicted fatal outcome with an odds ratio (OR) of 1.109 [95% confidence interval (95% CI): 1.082–1.137, *P* < 0.001; *Figure 1C*]. Using univariable logistic regression, a significant association with mortality was observed for D-dimer (OR = 1.058, 95% CI: 1.006–1.113, *P* = 0.028), PT (OR = 0.962, 95% CI: 0.948–0.977, *P* < 0.001), international normalized ratio (INR; OR = 2.904, 95% CI: 1.263–6.680, *P* = 0.012), and activated partial thrombin time (aPTT; OR = 1.034, 95% CI: 1.006–1.062, *P* = 0.017), showing an increased risk for mortality for patients with any deranged haemostatic biomarker. The determined predictive effects of each biomarker remained significant after addition of age to the logistic regression model (D-dimer: OR = 1.085, 95% CI: 1.015–1.158, *P* = 0.016; PT: OR = 0.965, 95% CI: 0.949–0.981, *P* < 0.001; INR: OR = 2.354, 95% CI: 1.096–5.057, *P* = 0.028; aPTT: OR = 1.039, 95% CI: 1.011–1.067, *P* = 0.006; *Figure 1C*). However, D-dimer and INR were not associated with mortality in the multivariable logistic regression model including age after multiple imputation (D-dimer: OR = 1.028, 95% CI: 0.972–1.087, *P* = 0.337; INR: OR = 1.592, 95% CI: 0.871–2.909, *P* = 0.130), while PT (OR = 0.979, 95% CI: 0.965–0.993, *P* = 0.005) and aPTT (OR = 1.028, 95% CI: 1.005–1.051, *P* = 0.015) remained significant in the final model. Ultimately, none of the evaluated biomarkers improved the prediction of fatal outcome based on age (age alone vs. age + D-dimer: *P* = 0.279, vs. age + PT: *P* = 0.083, vs. age + INR: *P* = 0.244, vs. age + aPTT: *P* = 0.121).

### 3.3 Markers of CAC and thromboinflammation are associated with disease severity in hospitalized COVID-19 patients and elevated in patients with poor outcome

In this cohort, D-dimer levels, H3Cit–DNA complexes, and cfDNA measured upon admission were associated with higher COVID-19 disease severity at admission (*P* = 0.046, *P* < 0.001, *P* = 0.041, respectively; Supplementary material online, *Figure S2A–C*). Further, patients with requirement of ICU treatment and non-survivors displayed increased D-dimer (uncomplicated vs. ICU: *P* = 0.014, uncomplicated vs. non-survivors: *P* < 0.001, ICU vs. non-survivors: *P* = 0.058; Supplementary material online, *Figure S2D*) and H3Cit–DNA complexes (uncomplicated vs. ICU: *P* = 0.001, uncomplicated vs. non-survivors: *P* = 0.013, ICU vs. non-survivors: *P* = 0.467; Supplementary material online, *Figure S2E*) at admission. A similar pattern was observed for cfDNA (uncomplicated vs. ICU: *P* = 0.018, uncomplicated vs. non-survivors: *P* = 0.151, ICU vs. non-survivors: *P* = 0.897; Supplementary material online, *Figure S2F*). There was no correlation of D-dimer levels and H3Cit–DNA complexes (*r* = −0.069, *P* = 0.531; Supplementary material online, *Figure S2G*), while cfDNA and D-dimer showed a weak, yet statistically significant correlation (*r* = 0.280, *P* = 0.010; Supplementary material online, *Figure S2H*).

### 3.4 CAC develops in COVID-19 patients with complicated hospitalization irrespective of disease severity upon admission

D-dimer dynamics during hospitalization were assessed and modelled using a mixed linear model approach. There was no difference in D-dimer increase between patients with mild or moderate COVID-19 and patients with severe or critical disease, as underlined by similar slopes for D-dimer increase (*P* = 0.440, Supplementary material online, *Figure S2I–J*). However, patients with severe and critical disease displayed increased D-dimer levels throughout the entire observation period (difference between D-dimer intercepts *P* < 0.001, Supplementary material online, *Figure S2K*).

Interestingly, the slopes of D-dimer showed a significant inclination in ICU patients and non-survivors whereas patients with uncomplicated disease did not display a relevant D-dimer increase during hospitalization (*P* < 0.001, *Figure 1D*, Supplementary material online, *Figure S3A and B*). However, there was no evidence for different slopes between patients who required ICU treatment and patients with fatal outcome (*P* = 0.230, Supplementary material online, *Figure S3C*).

H3Cit–DNA complexes in circulation decreased during hospitalization (*P* = 0.005; *Figure 1E*). There was no difference in H3Cit-DNA dynamics over time between patients with different outcomes (*P* = 0.123), while patients with ICU treatment and non-survivors displayed higher levels of H3Cit-DNA throughout the entire observational period when compared to patients with uncomplicated hospitalization (*P* < 0.001). In contrast, cfDNA dynamics varied according to outcome (*P* = 0.041, *Figure 1F*). In particular, patients with uncomplicated hospitalization and patients requiring ICU treatment showed a decrease over time, while non-survivors displayed a steady increase in cfDNA throughout hospitalization.

### 3.5 LMWH use is significantly associated with improved survival in hospitalized COVID-19 patients after adjusting for confounders

In order to evaluate which parameters influence hospital mortality, a multivariable Cox regression analysis was conducted (*Table 3*). Of note, the reported multivariable Cox regression model was carried out on the data set obtained after multiple imputations to account for missing data. Age, hypertension, cardiovascular diseases, chronic renal insufficiency, history of malignancy, requirement of oxygen at admission, oxygen saturation at admission, COVID-19 disease severity at admission, and use of LMWH were significantly associated with survival in univariable Cox regression. The multivariable model included all parameters being significant upon univariable Cox regression. Ultimately, older age, history of malignancy, and increasing COVID-19 disease severity increased the risk for mortality. In addition, LMWH remained significantly associated with reduced risk for mortality, as depicted by a hazard ratio (HR) of 0.561 (95% CI: 0.348–0.906). Additionally, a multivariable Cox regression model for adjustment to potential confounders and disparities in baseline demographics between survivors and non-survivors was fit. Here, we also included parameters that were not significant upon univariable Cox regression (Supplementary material online, *Table S4*). Again, use of LMWH was found to be independently associated with a decreased risk for mortality (HR = 0.539, 95% CI: 0.336–0.866). Of note, information on use of anticoagulants was missing in four patients. In total, 72.0% of patients were treated with LMWH from admission onwards, and 10.7% of patients were using non-vitamin-K oral anticoagulants (NOACs) (Supplementary material online, *Table S5*). Differences in baseline characteristics, disease severity, treatment, and outcome between anticoagulation subgroups were evaluated (Supplementary material online, *Table S5*). As use of NOAC was underrepresented in this cohort and not statistically associated with survival, the following analyses focused on LMWH. Importantly, only nine patients receiving LMWH were treated with therapeutic doses (i.e., ≥1.5 mg/kg daily), while the remaining 410 patients using LMWH received prophylactic doses.

**Table 3:** Cox regression analysis in multiple-imputation data set

Parameter	Univariable Cox regression			Multivariable Cox regression		
	HR	95% CI	*P*-value	HR	95% CI	*P*-value
Sex	1.125	0.720–1.757	0.650			
Age (years)	**1.075**	**1.055–1.096**	**<0.001**	**1.068**	**1.043–1.093**	**<0.001**
Comorbidities						
Current smoker	0.735	0.246–2.197	0.581			
Obesity (BMI > 25)	0.779	0.498–1.218	0.273			
Diabetes type II	1.317	0.835–2.077	0.237			
Hypertension	**1.838**	**1.156–2.920**	**0.010**	1.136	0.692–1.865	0.613
Cardiovascular disease (any)	**3.493**	**2.248–5.427**	**<0.001**	1.246	0.711–2.183	0.442
Chronic obstructive pulmonary disease	1.682	0.935–3.024	0.082			
Asthma	0.829	0.261–2.627	0.750			
Hypo-/hyperthyroidism	1.196	0.617–2.319	0.597			
Chronic renal insufficiency	**3.466**	**2.205–5.448**	**<0.001**	1.579	0.937–2.662	0.086
Chronic liver disease	0.689	0.252–1.883	0.468			
Malignancy	**1.978**	**1.163–3.364**	**0.012**	**2.054**	**1.184–3.561**	**0.010**
Symptoms at admission						
Asymptomatic	0.401	0.105–1.527	0.180			
Fatigue	1.054	0.690–1.610	0.806			
Cough	**0.634**	**0.414–0.972**	**0.036**	0.850	0.537–1.346	0.489
Fever	1.324	0.838–2.091	0.229			
Requirement of oxygen	**2.077**	**1.306–3.304**	**0.002**	1.639	1.001–2.682	0.050
Dyspnoea	1.205	0.788–1.842	0.389			
Diarrhoea	0.583	0.301–1.130	0.110			
Sore throat	0.185	0.027–1.259	0.085			
Nausea or vomiting	0.897	0.449–1.791	0.758			
Vital Signs at admission						
Temperature (°C)	0.882	0.698–1.114	0.291			
Pulse rate (beats per minute)	0.988	0.973–1.003	0.128			
Systolic arterial pressure (mmHg)	0.997	0.986–1.008	0.637			
Diastolic arterial pressure (mmHg)	0.989	0.968–1.010	0.290			
Respiratory rate (b.p.m.)	1.006	0.981–1.033	0.633			
SpO_2_ at ambient air (%)	**0.933**	**0.881–0.988**	**0.017**	1.038	0.957–1.125	0.368
COVID-19 classification at admission^a^	**1.654**	**1.303–2.099**	**<0.001**	**1.639**	**1.234–2.176**	**0.001**
Clinical characteristics						
Admission to ICU	1.161	0.738–1.827	0.517			
Invasive ventilation	1.196	0.722–1.982	0.487			
Anticoagulation						
LMWH	**0.413**	**0.270–0.634**	**<0.001**	**0.561**	**0.348–0.906**	**0.018**
NOAC	0.750	0.376–1.495	0.413			
Additional treatment						
Corticosteroids	0.611	0.366–1.019	0.059			
Remdesivir/Favipiravir	1.082	0.648–1.805	0.764			
Lopinavir/Ritonavir	0.475	0.219–1.031	0.060			
Camostat	0.044	0.001–1.829	0.101			

BMI, body mass index; CI, confidence interval; HR, hazard ratio; ICU, intensive care unit; LMWH, low-molecular-weight heparin; mmHg, millimetre mercury; NOAC, non-vitamin K oral anticoagulants; SpO_2_, peripheral capillary oxygen saturation.

a COVID-19 classification was performed according to the guidelines issued by the WHO in mild (fever <38°C, no dyspnoea, no pneumonia), moderate (fever, respiratory symptoms, pneumonia), severe (respiratory distress with respiratory rate ≥30 b.p.m., SpO_2_ < 93% at rest), and critical (respiratory failure with requirement of mechanical ventilation, requirement of ICU).

**Figure 2:**
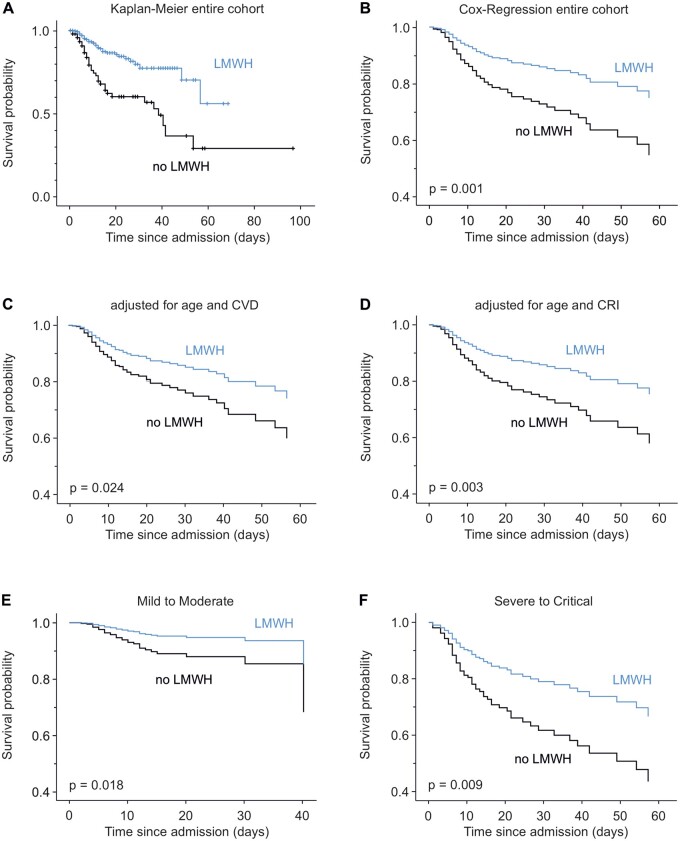
Anticoagulation with LMWH is associated with improved survival in COVID-19. (*A*) Kaplan–Meier curve showing survival for patients treated with LMWH (blue) and patients not using LMWH (black). Intersections represent censored patients. (*B–H*) Age-adjusted Cox regression plots show the effect of LMWH on survival for the entire cohort (*B*), after additional adjustment for CVD (*C*) or chronic renal insufficiency (CRI, *D*), as well as in patients with mild-to-moderate (*E*) or severe-to-critical (*F*) COVID-19 disease classification at admission (*N* = 582 in *A–D, N* = 365 in *E, N* = 217 in *F*).

After observing an association of LMWH with survival in a data set including multiple imputations, we aimed for description of these data on the original data set. Kaplan–Meier analysis according to status of LMWH intake is shown in *Figure 2A*. Corresponding Kaplan–Meier curves for each evaluated centre are shown in Supplementary material online, *Figure S4A–C*. In order to further evaluate the effect of LMWH on hospital mortality, additional Cox regression analyses including confounders and sub-groups were conducted. LMWH users showed improved survival upon age-adjusted Cox regression analysis (HR = 0.478, 95% CI: 0.312–0.733, *P* = 0.001, *Figure 2B*). Of note, we observed an increase in the proportion of LMWH treatment throughout the observed study period (Supplementary material online, *Figure S5*), which is due to the inclusion of anticoagulation in guidelines for treatment of COVID-19 in July 2020. In parallel, we observed a reduction of risk for mortality in this cohort, which was evaluated using Cox regression analysis for a time-dependent variable beginning with the inclusion of the first patient (HR = 0.991, 95% CI: 0.986–0.996, *P* = 0.001). Importantly, the association of LMWH with improved hospital survival was not affected by the time point of study inclusion, as the interaction term of LMWH × time of inclusion was not significantly associated with survival (HR = 1.013, 95% CI: 0.999–1.028, *P* = 0.075), while both the main effect of LMWH use and time of inclusion remained significant in the Cox regression analysis. The association of LMWH with survival further remained significant after including presence of cardiovascular diseases (HR = 0.586, 95% CI: 0.368–0.933, *P* = 0.024; *Figure 2C*) or chronic renal insufficiency (HR = 0.518, 95% CI: 0.336–0.800, *P* = 0.003; *Figure 2D*) as confounders for LMWH use. Additionally, as patients on anticoagulants frequently used further drugs (Supplementary material online, *Table S4*), multivariable Cox regression including treatment with corticosteroids, remdesivir/favipiravir, lopinavir/ritonavir, or camostat was computed and revealed a robust association of LMWH with hospital survival (Supplementary material online, *Figure S6*). Further, LMWH use was associated with improved survival in patients with mild-to-moderate disease (HR = 0.419, 95% CI: 0.204–0.862, *P* = 0.018; *Figure 2E*), as well as in patients with severe and critical COVID-19 at admission (HR = 0.488, 95% CI: 0.284–0.839, *P* = 0.009; *Figure 2F*). Respective data for use of NOAC can be found in Supplementary material online, *Figure S7*.

### 3.6 Anticoagulation is not associated with development of CAC, or reduction of markers of thromboinflammation

**Figure 3:**
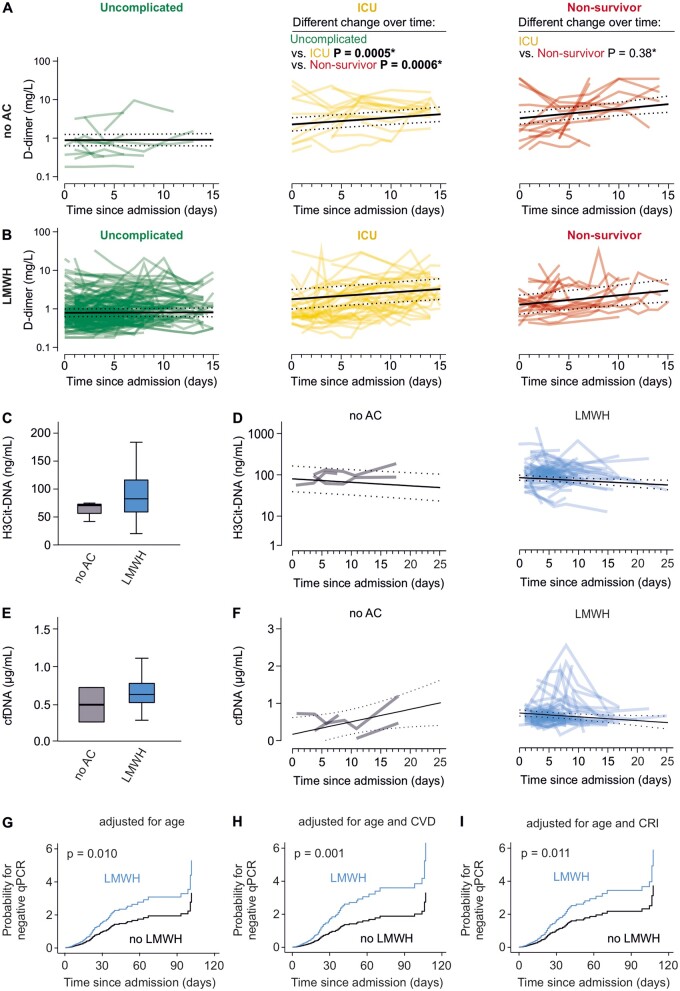
Anticoagulation has no effect on development of CAC and thromboinflammation while impacting viral persistence. (*A* and *B*) D-dimer time courses for patients with uncomplicated course of disease (green), those who were admitted to an ICU (yellow), and those who died (red) are plotted, and computed regression lines resulting from the applied mixed linear model are given including 95% CI (dotted lines) for patients without use of anticoagulation (no AC, *A*) and for patients treated with low-molecular heparin (LMWH, *B*). (*C*) Distribution of citrullinated histone 3 (H3Cit)–DNA complexes in circulation is illustrated for patients not using AC and patients treated with LMWH using box plots. Mann–Whitney *U* tests were used for comparison. (*D*) Time course of H3Cit–DNA complexes is modelled using a mixed linear model and shown for patients not using AC (grey) and patients treated with LMWH (blue). Computed regression lines for each group are given including 95% CI (dotted lines). (*E*) Distribution of cell-free DNA (cfDNA) in circulation is illustrated for patients not using AC and patients treated with LMWH using box plots. Mann–Whitney *U* tests were used for comparison. (*F*) Time course of cfDNA is modelled using a mixed linear model and shown for patients not using AC (grey) and patients treated with LMWH (blue). Computed regression lines for each group are given including 95% confidence interval (dotted lines). (*G–I*) Age-adjusted differences in viral persistence as estimated via Cox regression are shown in hazard plots comparing the probability of negative SARS-CoV-2 qPCR results over time for patients with and without use of LMWH in the entire cohort (*G*) and after additional adjustment for cardiovascular diseases (CVD, *E*) or chronic renal insufficiency (CRI, *F*) (*N* = 101 in *A, N* = 419 in *B, N* = 106 in *C–F, N* = 445 in *G–I*).

No difference in D-dimer dynamic was observed between patients with and without use of LMWH (*Figure 3A and B*). In particular, the effect of clinical course on D-dimer slopes was similar between patients treated with LMWH and patients who were not anticoagulated (*P* = 0.890). Likewise, use of NOAC was not associated with D-dimer increase in ICU patients and non-survivors (Supplementary material online, *Figure S8*). Due to the limited count of non-survivors taking NOAC, no conclusion can be made regarding these patients.

H3Cit–DNA complexes and cfDNA levels did not differ between patients with and without LMWH use at baseline (*P* = 0.360 and *P* = 0.173, respectively; *Figure 3C–F*). There was no difference in H3Cit-DNA dynamics over time between LMWH users and patients without LMWH use (*P* = 0.104; *Figure 3D*). Results obtained in patients using NOAC showed a similar pattern (Supplementary material online, *Figure S9A*). H3Cit-DNA concentration decreased over time (*P* = 0.009) irrespective of anticoagulation. Interestingly, cfDNA dynamics differed according to LMWH use (*P* = 0.039; *Figure 3F*), which was comparable between patients using NOAC (Supplementary material online, *Figure S9B*). In detail, cfDNA concentrations increased in patients without use of LMWH or NOAC, while a decrease over time of hospitalization was observed in patients using LMWH or NOAC.

### 3.7 Use of LMWH is associated with curtailed persistence of SARS-CoV-2 infection

Differences in viral persistence were assessed and compared according to use of anticoagulants. In total, information on viral persistence was available in 447 patients, with 60 patients (13.5%) not receiving anticoagulants, 337 (75.7%) receiving LMWH, and 48 patients (10.8%) receiving NOAC. Patients with LMWH use displayed a significantly increased probability for earlier negativity of SARS-CoV-2 qPCR upon age-adjusted Cox regression (HR = 1.593, 95% CI: 1.119–2.267, *P* = 0.010; *Figure 3G*), leading to a reduction in median time of viral persistence (median viral persistence no LMWH = 13 [IQR: 6-24] days vs. median viral persistence LMWH = 9 [IQR: 5–16] days, *P* = 0.009). Of note, the association of LMWH with curtailed viral persistence remained significant after additional adjustment for corticosteroids, antiviral agents, such as remdesivir/favipiravir or lopinavir/ritonavir, and camostat (Supplementary material online, *Figure S10*). Conversely, NOAC intake was not associated with curtailed SARS-CoV-2 persistence upon age-adjusted Cox regression (HR = 0.679, 95% CI: 0.410–1.123, *P* = 0.131; Supplementary material online, *Figure S11A*) and tended to be associated with prolonged viral persistence. Time of viral persistence did not differ between patients with and without NOAC intake [median viral persistence no NOAC = 10 (IQR: 5–17) days vs. median viral persistence NOAC = 12 (5–22) days, *P* = 0.331]. The beneficial effect of LMWH use was still observed after correction for cardiovascular diseases (HR = 1.914, 95% CI: 1.295–2.828, *P* = 0.001; *Figure 3H*) and after correction for chronic renal insufficiency (HR = 1.579, 95% CI: 1.108–2.251, *P* = 0.011; *Figure 3I*). Similarly, correction for these parameters did not alter the results obtained for NOAC which still did not associate with shortened viral persistence (Supplementary material online, *Figure S11B and C*). Importantly, the effect of LMWH on viral persistence was comparable in patients hospitalized at each evaluated centre (Supplementary material online, *Figure S12A–F*).

## 4. Discussion

In this observational multicentre investigation of 586 patients hospitalized for COVID-19 in Austria, we were able to show that the use of LMWH was associated with curtailed viral persistence in COVID-19 leading to a reduction of virus shedding of 4 days. LMWH use war further associated with increased survival and diminished circulating markers of cell death, while no differences in biomarkers of CAC development and thromboinflammation were observed between LMWH users and non-users.

Coagulation and the development of a hypercoagulable state play a central role in COVID-19 pathophysiology.^3^^,^^13–17^ In fact, CAC as depicted by D-dimer increase during hospitalization majorly evolved in patients requiring ICU and in non-survivors. While the mechanisms leading to CAC are a matter of ongoing investigations, increased D-dimer levels were postulated as a surrogate marker for presence of CAC, development of thrombotic complications, and ultimately as a predictor of mortality in COVID-19^4^^,^^18^ As previously reported, analysis of D-dimer and other haemostatic biomarkers revealed that these markers are of limited prognostic value for prediction of mortality in COVID-19.^19^ This indicates that more specific biomarkers are necessary to reliably predict patient outcome.

Thromboinflammation represents a central link between systemic hypercoagulability, respiratory failure, and mortality in COVID-19 patients, and NETs are frequently observed in CAC^20^^,^^21^ Investigation of post-mortem biopsies in COVID-19 patients showed occluding thrombi positive for citrullinated histone 3 as a specific marker for NETs not only in pulmonary tissue but also in kidney and cardiac tissue.^20^ In the present study, increased circulating markers of NET formation were observed in patients with higher COVID-19 disease severity at admission, which was paralleled by increased D-dimer levels, indicating that thromboinflammation might be a potential determinant of disease severity, as previously suggested.^21^ However, patients in the ICU and in the non-survivor sub-groups showed increased levels of H3Cit-DNA in circulation throughout the entire hospitalization. To our knowledge, this study is the first to assess markers of NET formation in a longitudinal approach, while previous studies focused on only one time point during disease onset. Intriguingly, we observed a general decrease in H3Cit-DNA regardless of outcome. This finding stands in clear contrast to the observed increase in D-dimer in patients requiring ICU treatment and non-survivors, suggesting a minor role of NET formation in CAC development or in later phases of COVID-19. In fact, there was no direct correlation of H3Cit-DNA and D-dimer in our study. Accordingly, while a role of NETs in COVID-19 pathophysiology could be validated, the contribution of thromboinflammation to CAC has to be questioned.

Importantly, we found that anticoagulation was associated with improved survival upon multivariable Cox regression analysis including age, cardiovascular diseases, chronic renal insufficiency, and concomitant treatment with other drugs affecting the course of COVID-19 as potential confounders, as well as in all evaluated sub-groups. This finding is in line with previous observational studies in COVID-19 patients, showing comparable effects of prophylactic and therapeutic anticoagulation on survival of COVID-19 patients.^6^^,^^7^^,^^17^ The use of anticoagulation and LMWH in particular entered the guidelines for treatment of COVID-19 in July 2020. Accordingly, we observed an increase in probability of LMWH use throughout the study period (Supplementary material online, *Figure S5*). Importantly, we could not evaluate an influence of the inclusion time point on the association of LMWH and improved survival, even though the biggest proportion of patients in the no-LMWH sub-group was included in the beginning of the study period. Intriguingly, in our cohort, anticoagulation with either LMWH or NOAC was not associated with altered dynamics of D-dimer indicating a minor effect of LMWH on CAC development. Alternatively, D-dimer levels in patients who received LMWH could be suppressed to levels observed in patients who did not receive LMWH, which was previously suggested by Blasi *et al*.^9^ Nonetheless, the observations made in this longitudinal approach do not allow to link improved CAC or altered haemostasis in LMWH treated COVID-19 patients to the observed improved survival. Accordingly, we aimed for investigation of potential off target effects of anticoagulation. We observed a decrease in the cell death marker cfDNA in patients using LMWH and NOAC during hospitalization. This is of specific interest, as an increase in cfDNA was only observed in non-survivors. Noticeably, a correlation of cfDNA and D-dimer could already be observed at baseline, potentially linking cell death to haemostatic derangements. Yet, these findings have to be interpreted with caution due to the observational character and the low number of patients not receiving anticoagulation in this evaluated sub-group. Nonetheless, our data are suggestive for a potential protective role of anticoagulants in COVID-19 beyond haemostasis. In this context, direct factor Xa inhibitors and heparin were shown to reduce oxidative stress and to yield anti-inflammatory properties, thereby potentially altering the inflammatory environment *in vivo* and affecting cell death in COVID-19.^22^^,^^23^ Preservation of vascular integrity via inhibition of endothelial cell heparinase or impairment of hepcidin formation and concomitant reduction of hyperferritinaemia might be other potential mechanisms by which LMWH exerts its beneficial effects.^24^^,^^25^

Importantly, we observed curtailed SARS-CoV-2 viral persistence upon qPCR in patients treated with LMWH, when compared to patients without anticoagulation or those using NOAC. These data provide exploratory clinical evidence compatible with a direct effect of LMWH on virus pathology, which was previously suggested in *in vitro* studies, where heparin was found to interfere with SARS-CoV-2 binding on ACE2 expressing cells, thereby limiting its infectivity.^26^ The effect on viral persistence was specific for LMWH in our analyses and increased odds for curtailed SARS-CoV-2 infection culminated in a median 4-day reduction of viral shedding. While direct interaction of LMWH with SARS-CoV-2 binding is one potential mechanism explaining the observed viral dynamics,^27^ the underlying study design does not allow to evaluate the exact pathomechanism responsible for these observations.

Notably, our study has certain limitations. In particular, the retrospective character of the study only allows to hypothesize a potential interaction of LMWH with SARS-CoV-2 which diminishes viral persistence. This limitation is further important for the interpretation of survival analyses, as the retrospective study design is associated with a notable amount of missing data. Accordingly, interventional studies are necessary to establish causality. In the light of the ongoing clinical trials, we aim to raise awareness for these potentially beneficial off-target effects of anticoagulants and encourage further research within this area. Moreover, we did not analyse effects of different doses of LMWH in our cohort, as only nine patients (2.2% of LMWH-treated patients) received therapeutic doses of LMWH and the statistical power for the respective analyses was not sufficient. Of note, previous reports were not able to assess a difference between prophylactic and therapeutic uses of LMWH in more than 4,000 patients.^6^ Further, recent data from interventional studies comparing therapeutic doses of LMWH to standard of care thromboprophylaxis showed a beneficial effect of high-dose treatments on hospital survival in non-critically ill patients, while critically ill patients did not benefit from these schemes.^28^^,^^29^ However, we cannot rule out that a difference in LMWH dosage might affect the data obtained for SARS-CoV-2 viral persistence in the present study. Ultimately, we want to point out that patients using NOAC were underrepresented in our cohorts, which renders the findings for this sub-cohort explorative and hypothesis generating. While we tried to take various possible confounders, for example comorbidities and age, into account, we cannot exclude that our data only reflect the situation in Austria and the virus mutations present.

Taken together, the present investigation confirms an association of anticoagulants with improved survival of COVID-19 patients in a large Central European Multicentre Cohort and suggests a beneficial effect of LMWH use on SARS-CoV-2 viral persistence. While the exact pathomechanisms underlying these observations cannot be investigated due to the retrospective observational study design, the present study encourages the evaluation of viral persistence in randomized controlled trials assessing the effect of LMWH in COVID-19 patients in order to establish a causal relation of the presented findings. Limiting viral persistence, thereby shortening hospitalization and contagiousness is a relevant aspect during this pandemic.

## Supplementary material

Supplementary material is available at *Cardiovascular Research* online.

## Authors’ contributions

D.P. collected and analysed the data, prepared the figures, and wrote the manuscript; S.H. performed statistical analyses, prepared the figures, and wrote the manuscript; W.C.S., J.S., A.P., A.S., K.K., D.A., T.S., F.F., H.H., E.P., P.K., B.R., M.T.T., P.G., C.F., C.S., T.S., and M.K. collected and analysed the data; I.P., C.B., P.S., G.W., R.B.W., H.J.F.S., B.J., and A.Z. provided resources and interpreted the data; A.A. conceived the study, analysed and interpreted the data, and wrote the manuscript.

**Conflict of interest:** none declared.

## Funding 

This work is part of the ACOVACT study of the Medical University of Vienna and is financially supported by the Austrian Federal Ministry of Education, Science and Research, the Medical-Scientific Fund of the Mayor of Vienna (COVID024), and the Austrian Science Fund (P32064; SFB-54).

## Data availability

The data presented in this article are represented in the main text and in the article’s Supplementary material online. Additional information can be shared after reasonable request to the corresponding author.

## Supplementary Material

cvab308_Supplementary_Data

## Notes

Time for primary review: 19 days

## Notes

Translational perspective The data gathered in this retrospective multicentre observational study could confirm an association of improved survival and anticoagulation and could link LMWH use to improved biomarkers of cell death and curtailed persistence of SARS-CoV-2. This encourages the use of this drug in patients without known contraindications in case this effect can be consolidated in randomized controlled trials. Currently performed prospective trials using LMWH in COVID-19 should focus on viral persistence in order to explore an additional field of application for LMWH during this pandemic.
