# Supplementary material for: Low-molecular-weight heparin use in coronavirus disease 2019 is associated with curtailed viral persistence: a retrospective multicentre observational study
Source: Cardiovasc Res. 2021 Oct 5;117(14):2807–20. doi: 10.1093/cvr/cvab308 (PMC8500043; doi:10.1093/cvr/cvab308)
Supplement: cvab308_Supplementary_Data [file cvab308_supplementary_data.docx]

**Online Supplement**

Low molecular weight heparin use in COVID-19 is associated with curtailed viral persistence– a retrospective multicenter observational study

David Pereyra MD^1,2^, Stefan Heber MD, PhD^3^, Waltraud C. Schrottmaier PhD^1^, Jonas Santol^2^, Anita Pirabe MSc^1^, Anna Schmuckenschlager MSc^1^, Kerstin Kammerer^1^, Daphni Ammon BSc^2^, Thomas Sorz^2^, Fabian Fritsch^2^, Hubert Hayden MSc^4^, Erich Pawelka MD^5^, Philipp Krüger^1,2^, Benedikt Rumpf^2,5^, Marianna T. Traugott MD^5^, Pia Glaser MSc^6^, Christa Firbas MD^7^, Christian Schörgenhofer MD^7^, Tamara Seitz MD^5^, Mario Karolyi MD^5^, Ingrid Pabinger Prof. MD^6^, Christine Brostjan Prof. PhD^4^, Patrick Starlinger Prof. MD, PhD^2^, Günter Weiss Prof. MD^8^, Rosa Bellmann-Weiler Prof. MD^8^, Helmut J.F. Salzer MD^9^, Bernd Jilma Prof. MD^7^, Alexander Zoufaly MD^5^, Alice Assinger Prof. PhD^1,†^

^1^ Department of Vascular Biology and Thrombosis Research, Center of Physiology and Pharmacology, Medical University of Vienna, Vienna, Austria

^2^ Department of Surgery, Division of Visceral Surgery, Medical University of Vienna, General Hospital Vienna, Vienna, Austria

^3^ Institute of Physiology, Centre of Physiology and Pharmacology, Medical University of Vienna, Vienna, Austria

^4^ Department of Surgery, Division of Vascular Surgery, Medical University of Vienna, General Hospital Vienna, Vienna, Austria

^5^ Department of Medicine IV, Kaiser Franz Josef Hospital, Vienna, Austria

^6^ Department of Medicine I, Medical University of Vienna, General Hospital Vienna, Vienna, Austria

^7^ Department of Clinical Pharmacology, Medical University of Vienna, General Hospital Vienna, Vienna, Austria

^8^ Department of Internal Medicine II, Medical University of Innsbruck, Innsbruck, Austria

^9^ Department of Pulmonology, Kepler University Hospital and Johannes Kepler University, Linz, Austria.

^†^ Correspondence: Alice Assinger, PhD

Medical University of Vienna,

Center of Physiology and Pharmacology,

Institute for Vascular Biology and Thrombosis Research

Schwarzspanierstraße 17, 1090 Vienna, Austria

E-mail: alice.assinger@meduniwien.ac.at

Telephone: +43 1 40160 31405

Original Research

Short Title: Low molecular weight heparin in COVID-19

**Supplemental Figures**

**
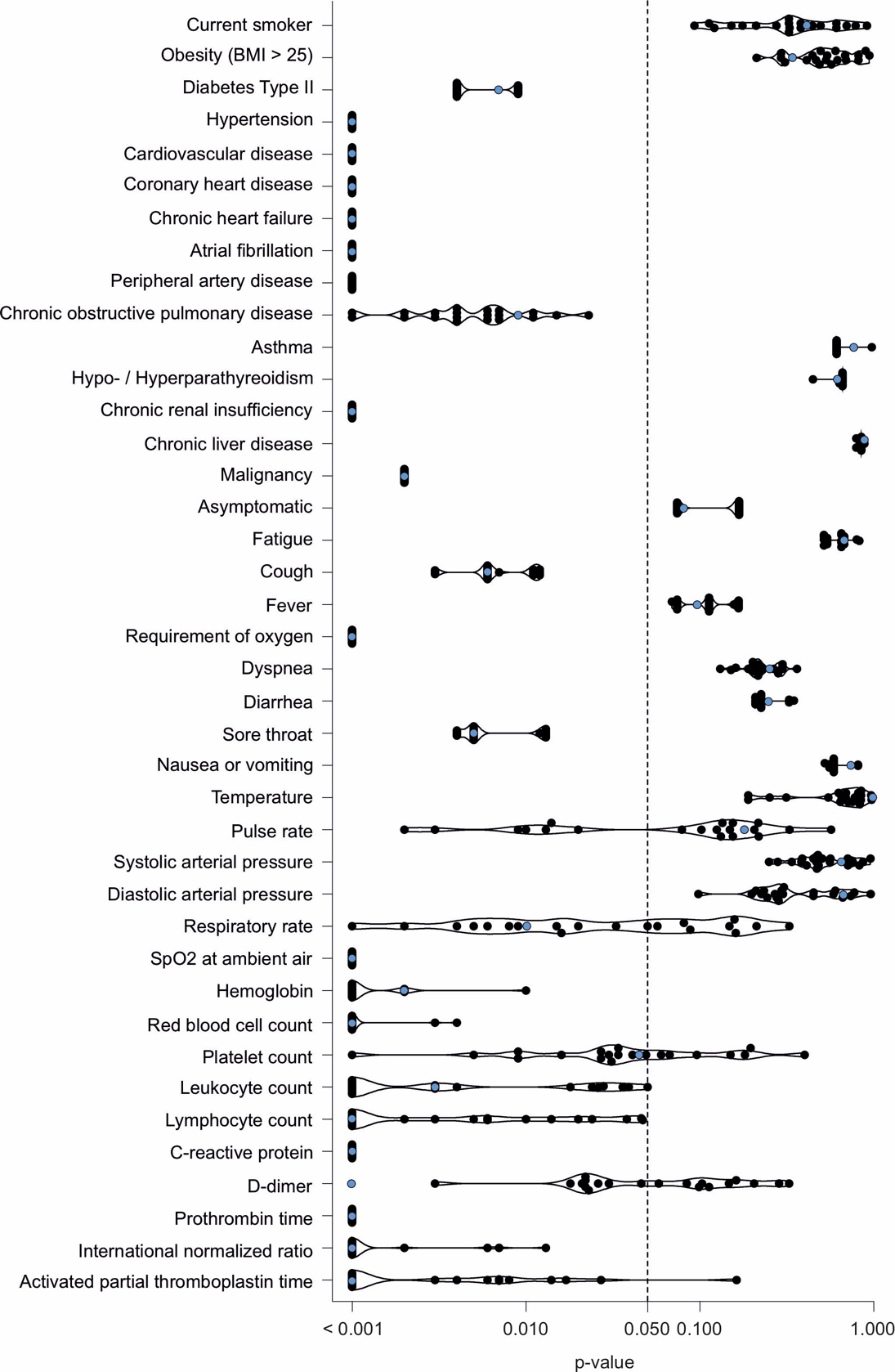
**

**Supplemental Figure 1.**

Multiple imputation was conducted in order to account for missing data. The respective p-values for the comparison of each variable between survivors and non-survivors are given for 20 imputed data sets. P-values obtained from the original data set are visualized in blue. The dotted line marks p=0.050.


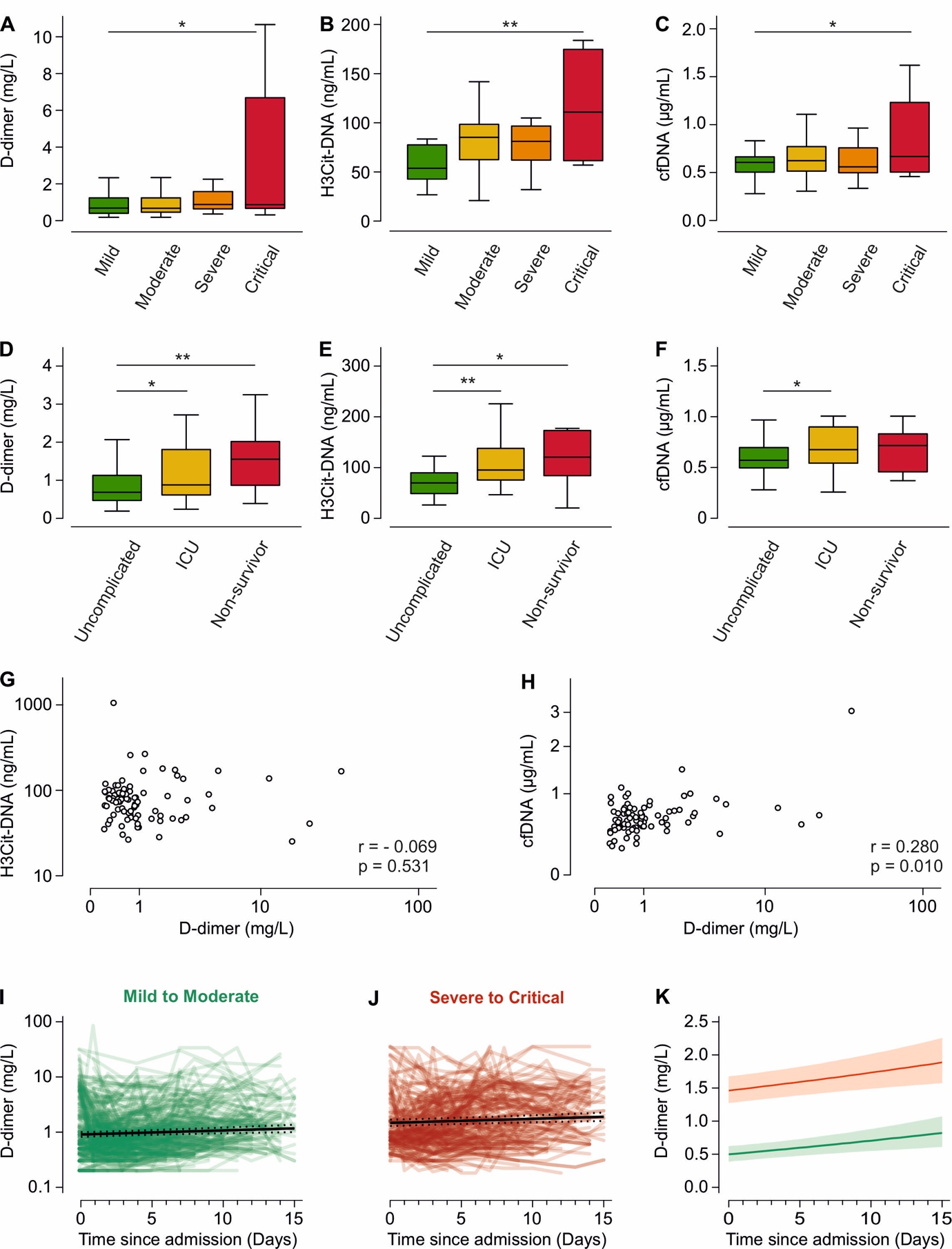


**Supplemental Figure 2.**

**A-F)** Box plots illustrating the distribution of D-dimer **(A, D)**, citrullinated histone 3 – DNA (H3Cit-DNA) **(B, E)**, and cell-free DNA (cfDNA) **(C, F)** in plasma are shown according to COVID-19 classification at admission **(A-C)** and according to outcome **(D-F)**. Mann-Whitney-U tests were used for this comparison. Accordingly, D-dimer increases with higher COVID-19 disease severity (median D-dimer mild = 0.69 [IQR: 0.38-1.24] mg/L, median D-dimer moderate = 0.68 [IQR: 0.46-1.31] mg/L, median D-dimer severe = 0.88 [IQR: 0.64-1.59] mg/L, median D-dimer critical = 0.87 [IQR: 0.66-8.70] mg/L, p=0.046, **A**), which was similar for H3Cit-DNA complexes (median H3Cit-DNA mild = 52.96 [IQR: 41.72-78.97] ng/mL, median H3Cit-DNA moderate = 84.37 [IQR: 57.75-98.72] ng/mL, median H3Cit-DNA severe = 80.29 [IQR: 59.10-99.55] ng/mL, median H3Cit-DNA critical = 110.08 [IQR: 59.84-175.50] ng/mL, p<0.001, **B**) and cfDNA (median cfDNA mild = 0.61 [IQR: 0.49-0.66] µg/mL, median cfDNA moderate = 0.62 [IQR: 0.50-0.77] µg/mL, median cfDNA severe = 0.558 [IQR: 0.50-0.77] µg/mL, median cfDNA critical = 0.67 [IQR: 0.50-1.35] µg/mL, p=0.041, **C**). Patients with complicated hospitalization and non-survivors further showed increased D-dimer (median D-dimer uncomplicated = 0.68 [IQR: 0.46-1.18] mg/dL vs median D-dimer ICU = 0.87 [IQR: 0.60-1.89] mg/dL, p=0.014; uncomplicated vs median D-dimer non-survivors = 1.55 [IQR: 0.86-2.04] mg/dL, p<0.001; ICU vs non-survivors p=0.058; **D**), H3Cit-DNA (median H3Cit-DNA uncomplicated = 69.47 [IQR: 48.41-89.81] ng/mL vs median H3Cit-DNA ICU = 94.98 [IQR: 74.85-139.30] ng/mL, p=0.001; uncomplicated vs median H3Cit-DNA non-survivors = 120.28 [IQR: 80.14-173.87] ng/mL, p=0.013; ICU vs non-survivors p=0.467; **E**) and cfDNA (median cfDNA uncomplicated = 0.57 [IQR: 0.49-0.69] µg/mL vs median cfDNA ICU = 0.67 [IQR: 0.53-0.91] µg/mL, p=0.018; uncomplicated vs median cfDNA non-survivors = 0.71 [IQR: 0.44-0.87] µg/mL, p=0.151; ICU vs non-survivors p=0.897; **F**). **G-H)** Dot plot illustrating the association between D-dimer and H3Cit-DNA **(G)** as well as D-dimer and cfDNA **(H)** at admission. Correlation was estimated using non-parametric Spearman correlation. **I-K)** D-dimer time courses for patients with mild or moderate (green) **(I)** and for patients with severe or critical COVID-19 classification at admission (red) **(J)** are plotted and computed regression lines resulting from the applied mixed linear model are given including 95% confidence interval (dotted lines). **K)** Regression lines and 95% confidence interval (faded areas) for both subgroups are compared. (N=586 for evaluation of D-dimer, N=106 for evaluation of H3Cit-DNA and cfDNA; *p<0.05, **p<0.001)

**
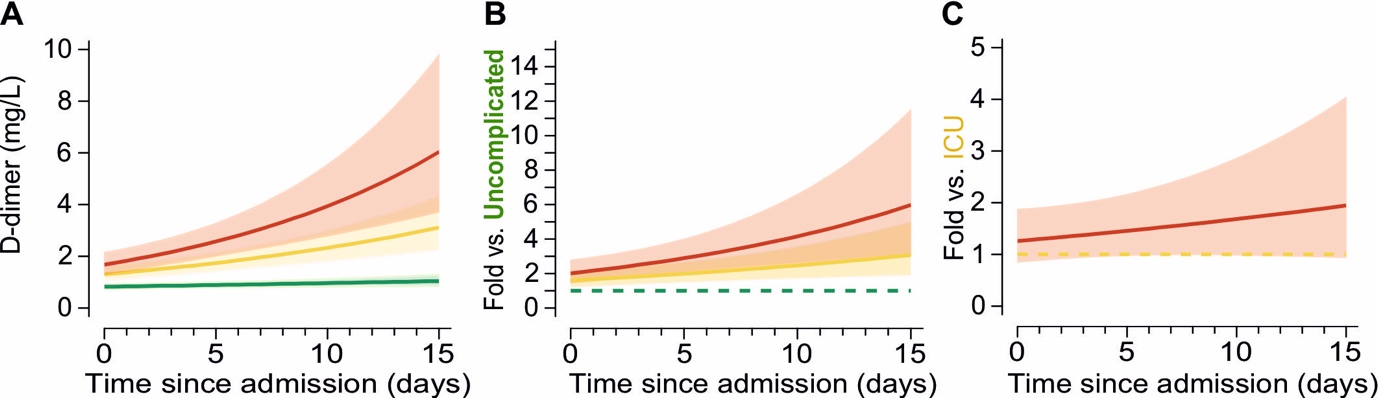
**

**Supplemental Figure 3.**

**A)** Least square geometric means with 95% confidence intervals for visualization of the time courses of D-dimer for patients with uncomplicated course (green), patients requiring intensive care unit (ICU, yellow) and non-survivors (red). **B)** Estimated difference between D-dimer levels of patients with uncomplicated disease and the others. The 95% confidence intervals not including the reference group indicate statistically significant differences throughout the whole period of 15 days. **C)** No significant difference in D-dimer time courses between patients admitted to ICU and those who died, as visualized by the confidence interval including the reference line. The transparent areas represent the respective 95% confidence intervals. (N=586 in A-C)

**
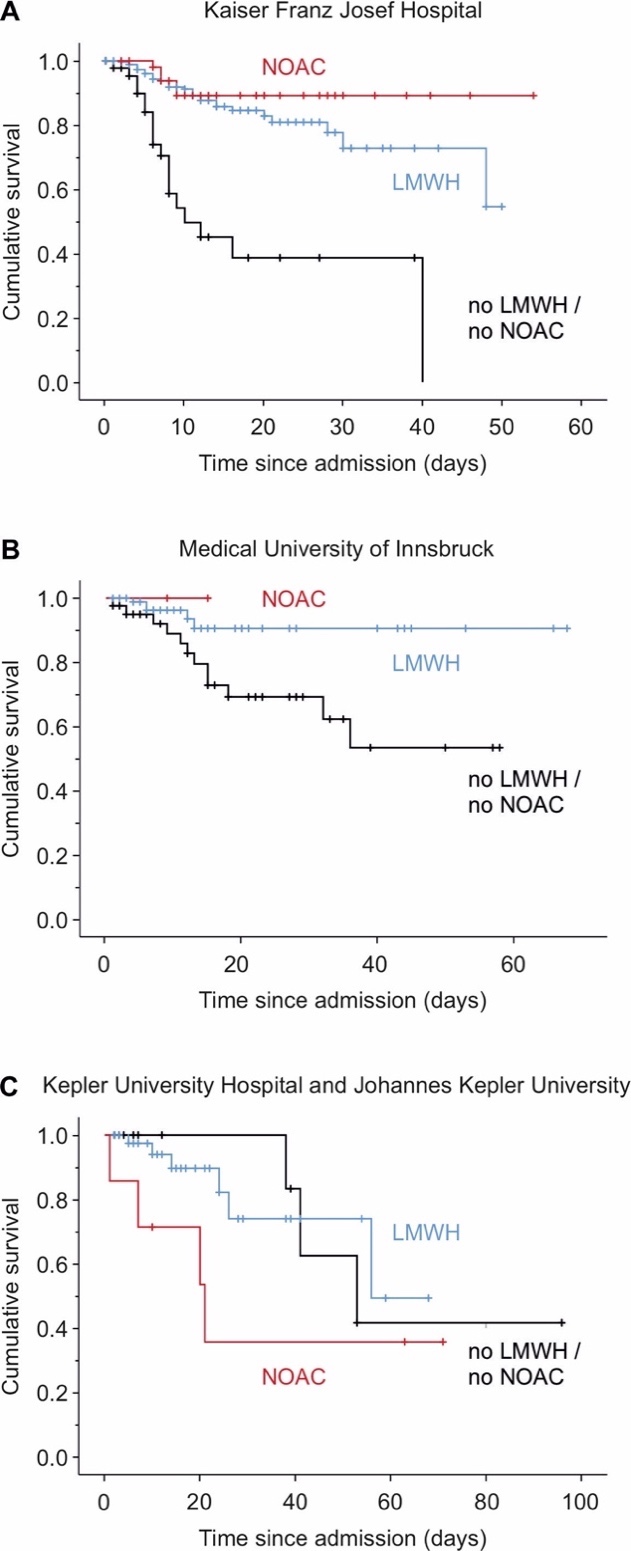
**

**Supplemental Figure 4.**

**A-C)** Kaplan-Meier curves showing survival for patients treated with low molecular weight heparin (LMWH, blue), non-vitamin K antagonist oral anticoagulants (NOAC, red) and patients receiving neither of these anticoagulants (black) are shown for each evaluated center, namely Kaiser Franz Josef Hospital **(A)**, Medical University of Innsbruck **(B)** and Kepler University Hospital and Johannes Kepler University **(C)**. Intersections represent censored patients. (N=375 in A-B, N=143 in C-D, N=64 in E-F)

**
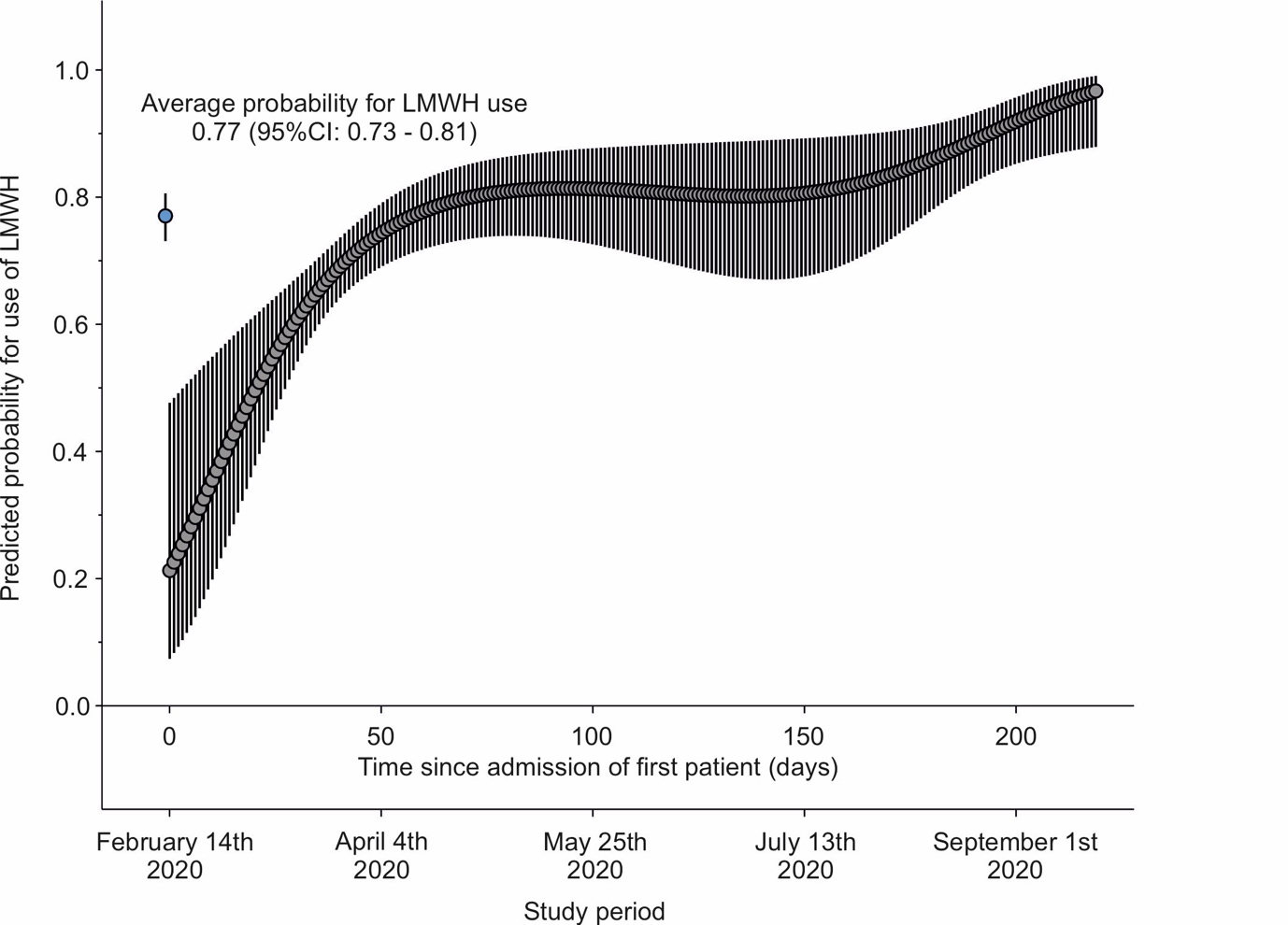
**

**Supplemental Figure 5.**

Predicted probability of low molecular weight heparin (LMWH) use across study period was estimated using a generalized linear model. Estimates for each consecutive day are shown with respective 95% confidence intervals (95%CI). Average probability for LMWH use in the study is shown in blue. (N=582)

**
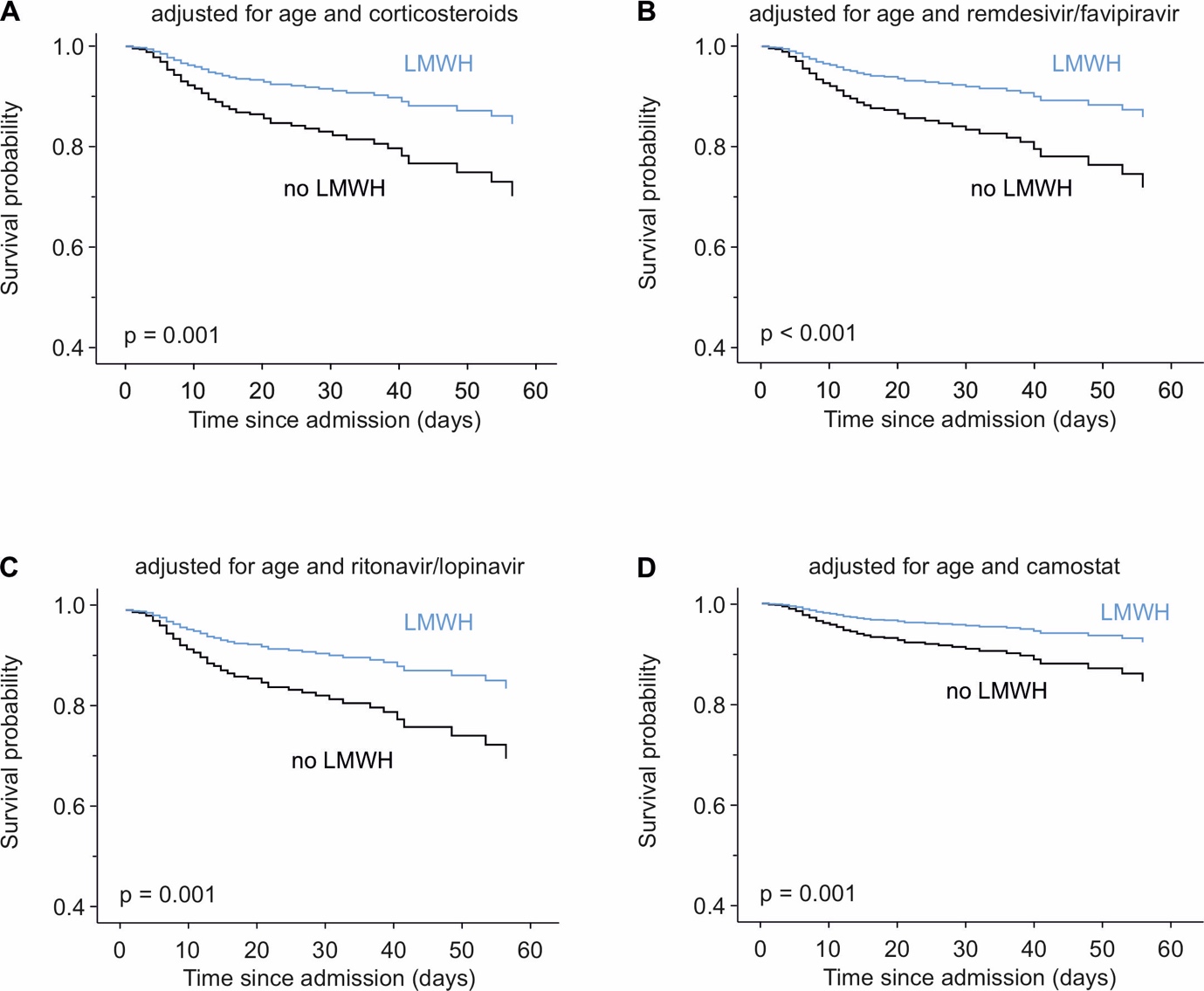
**

**Supplemental Figure 6.**

**A-D)** Age-adjusted Cox-regression plots show the effect of LMWH on survival for the entire cohort with additional adjustment for treatment with corticosteroids (A), remdesivir/favipiravir (B), lopinavir/ritonavir (C), and camostat (D). (N=581 in A, N=582 in B-D)

**
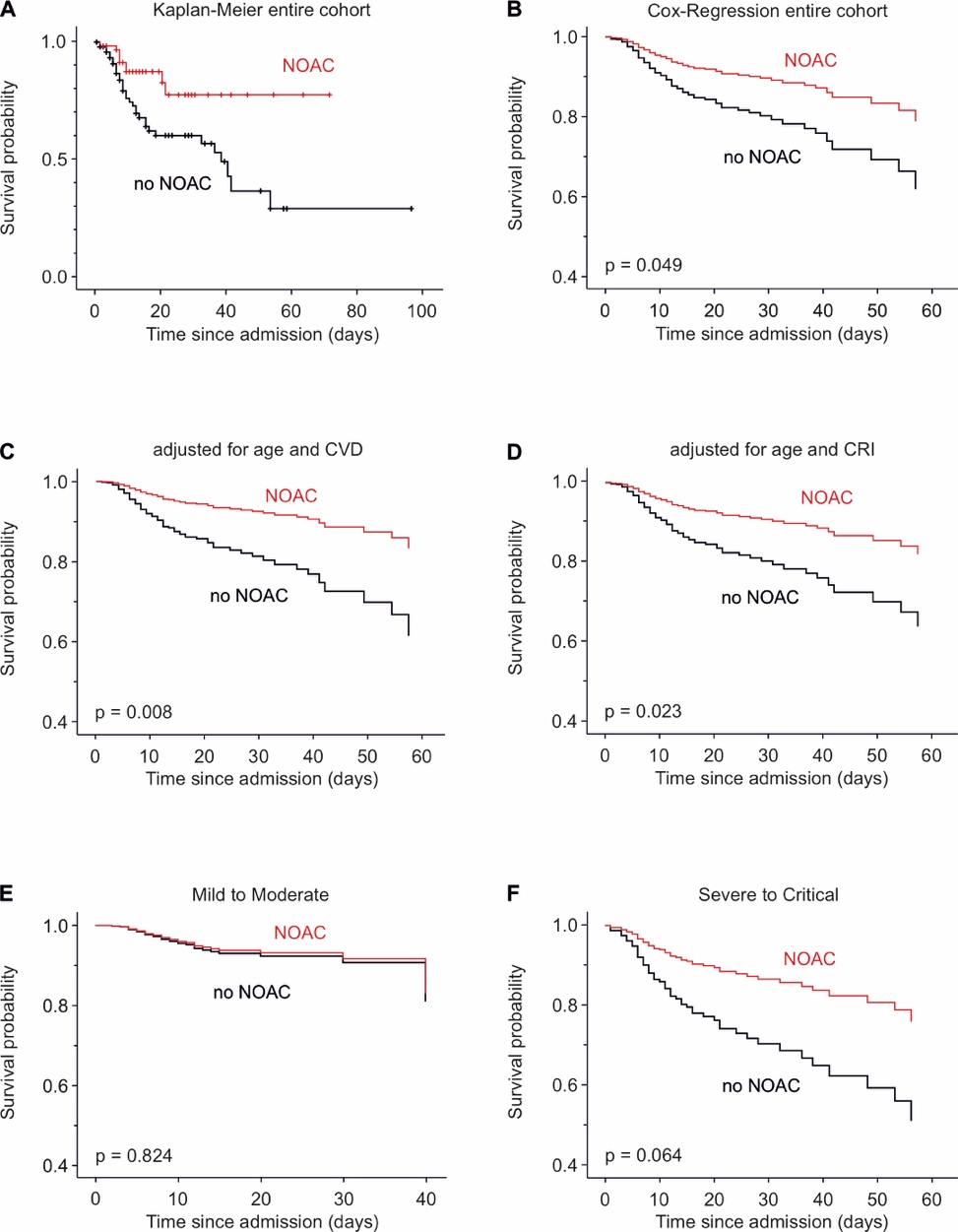
**

**Supplemental Figure 7.**

**A)** Kaplan-Meier curve illustrating the difference in survival between patients using non-vitamin K antagonist oral anticoagulants (NOAC) and patients without use of NOAC. **B-F)** Age-adjusted Cox-regression plots show the effect of NOAC on survival for the entire cohort **(B)**, with additional adjustment for cardiovascular diseases (CVD, **C**) or chronic renal insufficiency (CRI, **D**), as well as for patients with mild to moderate **(E)** and severe to critical disease classification **(F)**. (N=586 in A-D, N=366 in E, N=220 in F)

**
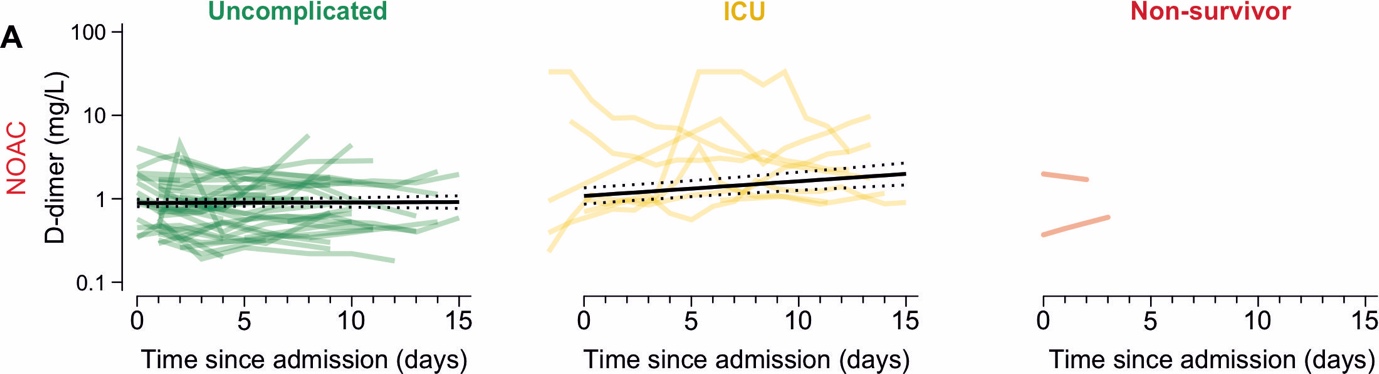
**

**Supplemental Figure 8.**

**A)** Time courses of D-dimer is shown for patients with uncomplicated course of disease (green), those who were admitted to an intensive care unit (ICU, yellow), and for non-survivors (red). Time courses were modeled using mixed linear models, lines with dotted error bands represent least squared means of log-transformed D-dimer values with 95% confidence intervals. (N=62 in A-C)

**
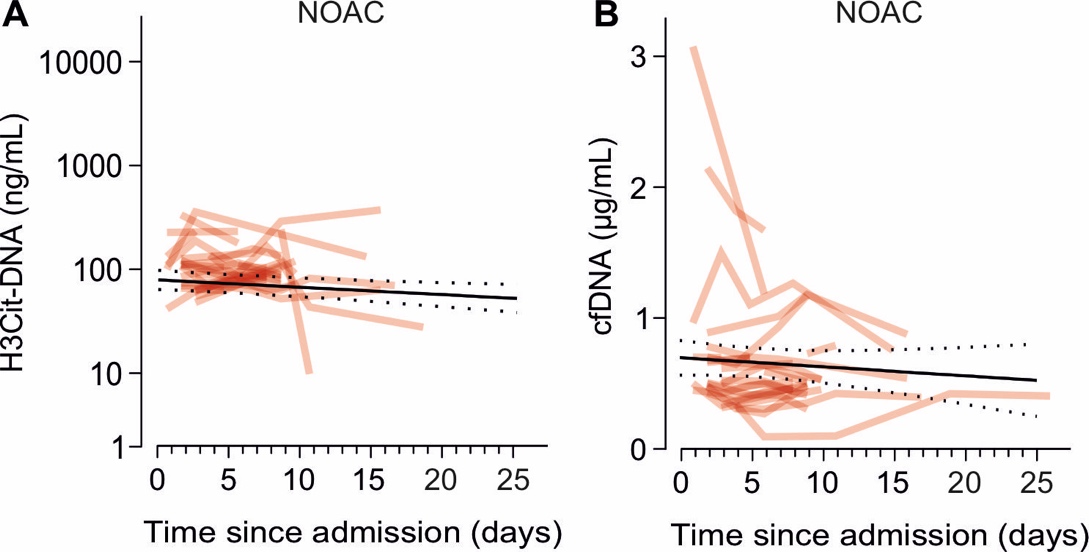
**

**Supplemental Figure 9.**

**A-B)** Time courses of citrullinated histone 3-DNA (H3Cit-DNA) **(A)** and cell-free DNA (cfDNA, **B**) for patients are plotted for patients using non-vitamin K antagonist oral anticoagulants (NOAC) and computed regression lines for each group are given including 95% confidence interval (dotted lines). (N=30 in A-B)

**
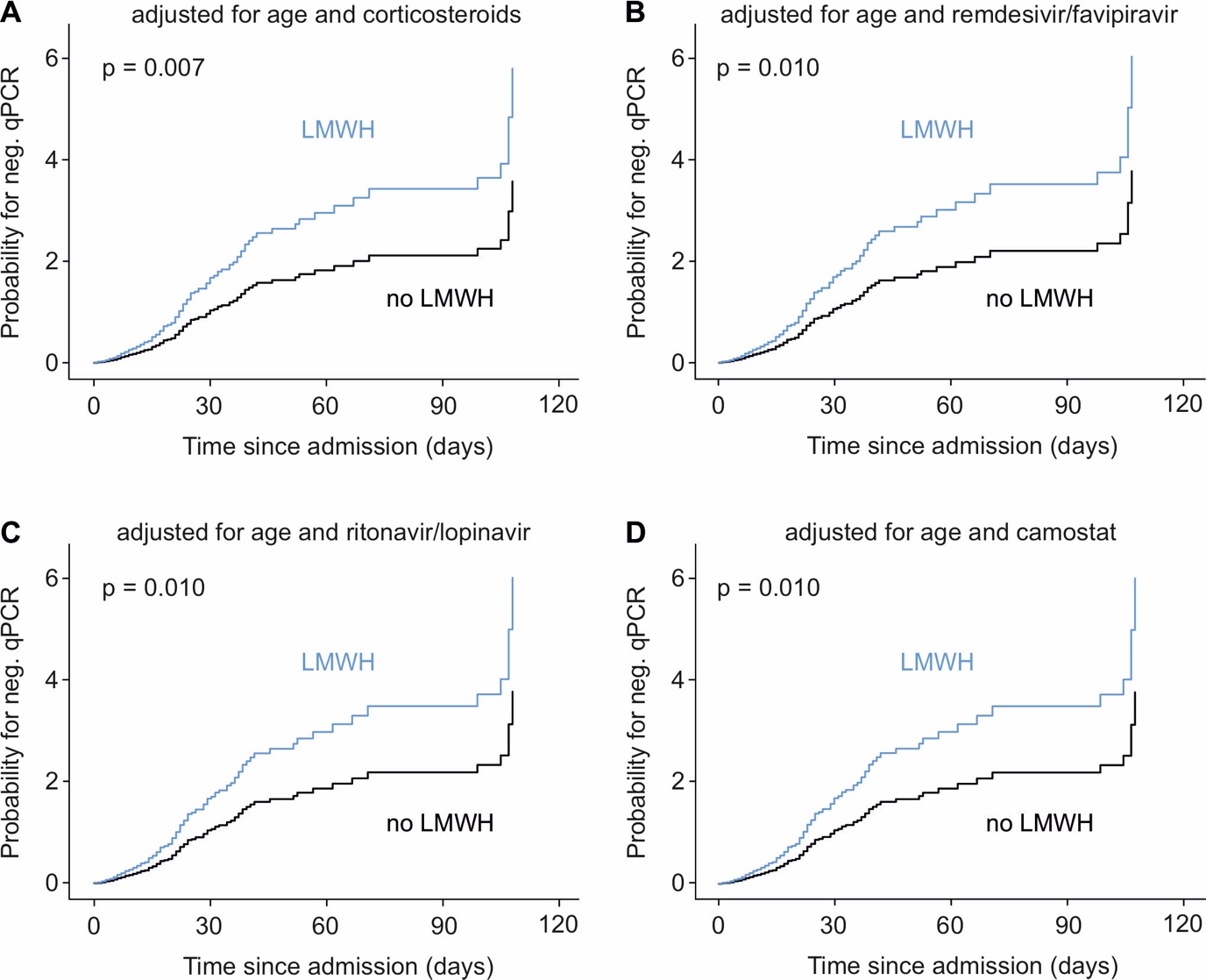
**

**Supplemental Figure 10.**

**A-C)** Differences in viral persistence as estimated via Cox-regression analysis are shown in Hazard plots comparing the probability of negative quantitative polymerase chain reaction (qPCR) results over time for patients with and without use of low molecular weight heparin (LMWH) adjusted for age and treatment with corticosteroids (A), remdesivir/favipiravir (B), lopinavir/ritonavir (C), and camostat (D). (N=445 in A, N=444 in B-D)


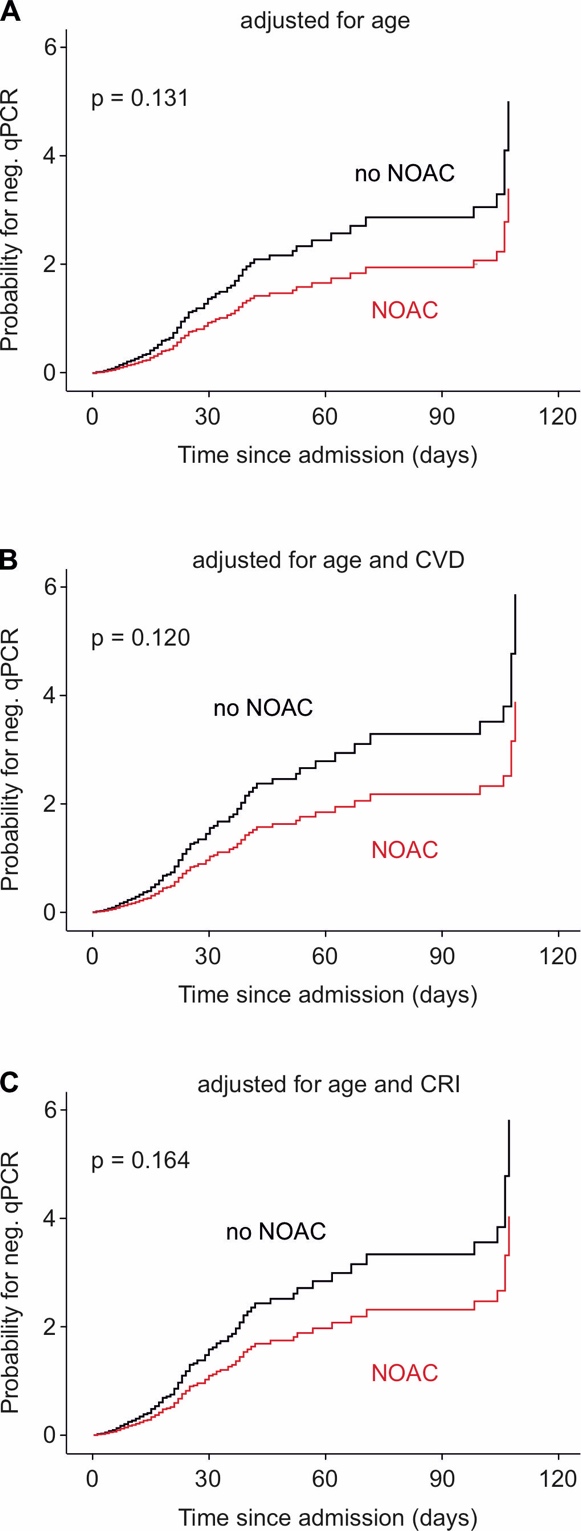


**Supplemental Figure 11.**

**A-C)** Differences in viral persistence as estimated via Cox-regression analysis are shown in Hazard plots comparing the probability of negative quantitative polymerase chain reaction (qPCR) results over time for patients with and without use of non-vitamin K antagonist oral anticoagulants (NOAC) with age adjustment in the entire cohort **(A)**, as well as after additional adjustment for cardiovascular diseases (CVD, **B**) and chronic renal insufficiency (CRI, **C**). (N=445 in A-C)

**
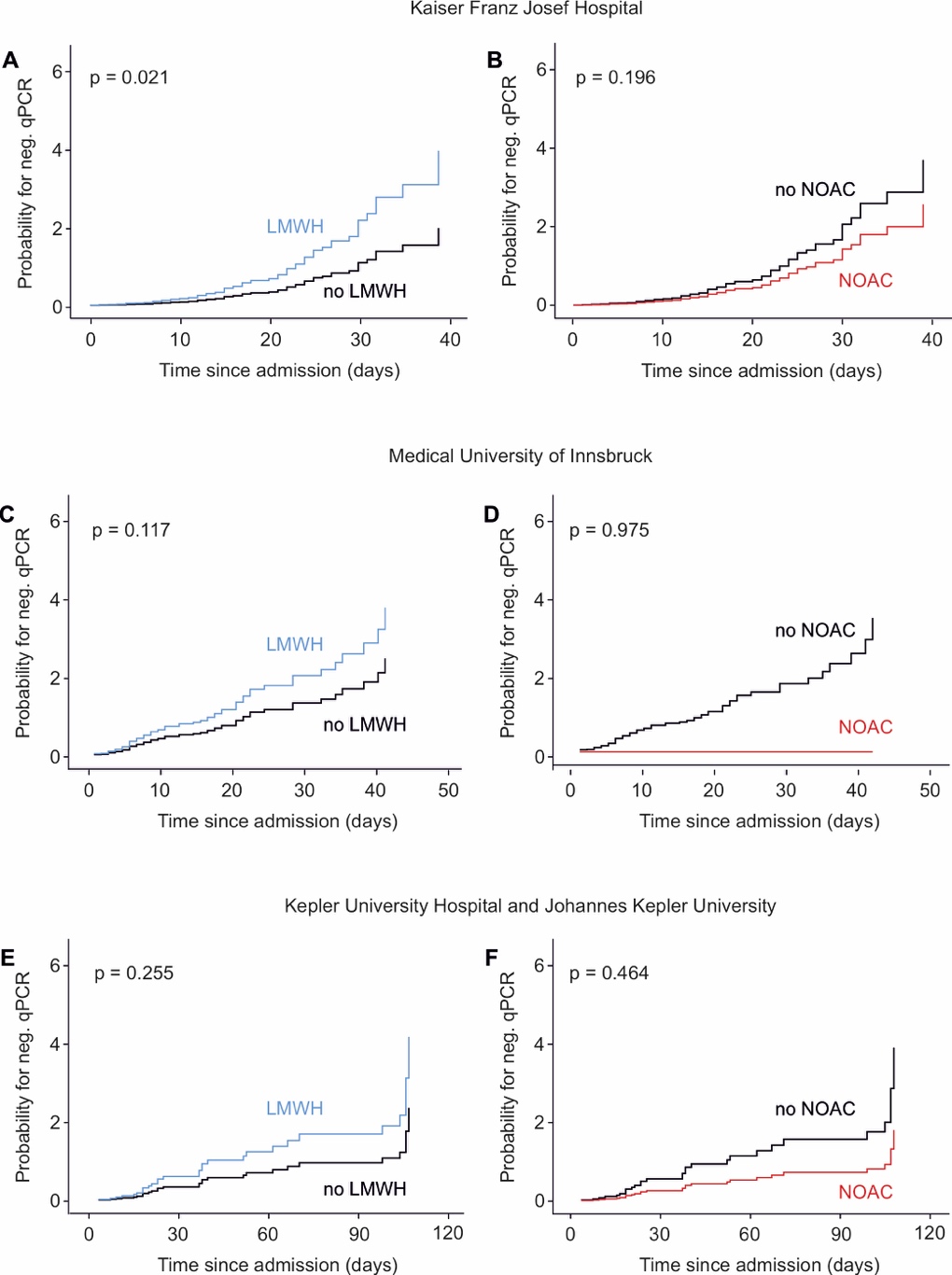
**

**Supplemental Figure 12.**

**A-F)** Differences in viral persistence as estimated via Cox-regression are shown in Hazard plots comparing the probability of negative quantitative polymerase chain reaction (qPCR) results over time for patients with and without use of low molecular heparin (LMWH) **(A, C, E)** or with and without intake of non-vitamin K antagonist oral anticoagulants (NOAC) **(B, D, F)** for each evaluated center, namely Kaiser Franz Josef Hospital **(A, B)**, Medical University of Innsbruck **(C, D)** and Kepler University Hospital and Johannes Kepler University **(E, F)**. (N=291 in A-B, N=108 in C-D, N=46 in E-F)

**Supplemental Figure 13.**

STROBE check list – page 1

**Supplemental Figure 14.**

STROBE check list – page 2

**Supplemental Tables**

| **Supplemental Table 1. Patient demographics in plasma cohort** | | |
| --- | --- | --- |
|  | **Missing Data** | **Plasma Cohort (N=106)** |
| **Parameter** | **N** | **N (%) / Median (IQR)** |
| **Sex** | 0 |  |
| Female |  | 36 (34.0%) |
| Male |  | 70 (66.0%) |
| **Age (years)** | 0 | 62 (49 – 77) |
| **Comorbidities** |  |  |
| Current smoker | 38 | 7 (6.6%) |
| Obesity (BMI > 25) | 14 | 47 (44.3%) |
| Diabetes type II | 0 | 30 (28.3%) |
| Hypertension | 1 | 62 (58.5%) |
| Cardiovascular disease (any) | 0 | 30 (28.3%) |
| Coronary heart disease | 0 | 15 (14.2%) |
| Chronic heart failure | 0 | 9 (8.5%) |
| Atrial fibrillation | 0 | 12 (11.3%) |
| Peripheral arterial disease | 0 | 5 (4.7%) |
| Chronic obstructive pulmonary disease | 0 | 12 (11.3%) |
| Asthma | 0 | 4 (3.8%) |
| Hypo- / Hyperthyroidism | 1 | 11 (10.4%) |
| Chronic renal insufficiency | 0 | 13 (12.3%) |
| Chronic liver disease | 1 | 6 (5.7%) |
| Malignancy | 0 | 12 (11.3%) |
| **Symptoms at admission** |  |  |
| Asymptomatic | 1 | 11 (10.4%) |
| Fatigue | 1 | 61 (57.5%) |
| Cough | 1 | 73 (68.9%) |
| Fever | 1 | 63 (59.4%) |
| Requirement of oxygen | 1 | 60 (56.6%) |
| Dyspnea | 1 | 53 (50.0%) |
| Diarrhea | 1 | 13 (12.3%) |
| Sore throat | 2 | 10 (9.4%) |
| Nausea or vomiting | 1 | 9 (8.5%) |
| **Vital Signs at admission** |  |  |
| Temperature (°C) | 67 | 37.9 (36.6 – 38.5) |
| Pulse rate (beats per minute) | 30 | 86 (80 – 99) |
| Systolic arterial pressure (mmHg) | 25 | 130 (120 – 143) |
| Diastolic arterial pressure (mmHg) | 27 | 80 (70 – 82) |
| Respiratory rate (breaths per minute) | 81 | 20 (15 – 24) |
| SpO_2_ at ambient air (%) | 36 | 95 (92 – 96) |
| **COVID-19 classification at admission^†^** | 0 |  |
| Mild |  | 19 (17.9%) |
| Moderate |  | 46 (43.4%) |
| Severe |  | 29 (27.4%) |
| Critical |  | 12 (11.3%) |
| **Medication at admission** | 0 |  |
| LMWH |  | 73 (68.9%) |
| NOAC |  | 30 (28.3%) |
| **Clinical Characteristics** |  |  |
| Total hospitalization (days) | 0 | 13 (9 – 23) |
| Admission to ICU | 0 | 33 (31.1%) |
| Invasive ventilation | 0 | 12 (11.3%) |
| Non-survivors | 0 | 11 (10.4%) |
| IQR = interquartile range, BMI = body mass index, mmHg = millimeter mercury, SpO_2_= peripheral capillary oxygen saturation, COVID-19 = Corona virus disease 2019, LMWH = low molecular weight heparin, NOAC = non-vitamin K anticoagulants, ICU = intensive care unit  **^†^** COVID-19 classification was performed according to the guidelines issued by the World Health Organization in mild (fever <38°C, no dyspnea, no pneumonia), moderate (fever, respiratory symptoms, pneumonia), severe (respiratory distress with respiratory rate ≥30 breaths per minute, SpO_2_ < 93% at rest) and critical (respiratory failure with requirement of mechanical ventilation, requirement of ICU) | | |

| **Supplemental Table 2. Patient demographics according to institution** | | | | | | | |
| --- | --- | --- | --- | --- | --- | --- | --- |
|  | **Missing Data** | **KFJ**  **(N=379)** | **Missing Data** | **MUI**  **(N=143)** | **Missing Data** | **KUH-JKU**  **(N=64)** |  |
| **Parameter** | **N** | **N (%)**  **Median**  **(IQR)** | **N** | **N (%)**  **Median**  **(IQR)** | **N** | **N (%)**  **Median**  **(IQR)** | **p-value** |
| **Sex** | 0 |  | 0 |  | 0 |  | 0.408 |
| Female |  | 150 (39.6%) |  | 53 (37.1%) |  | 30 (46.9%) |  |
| Male |  | 229 (60.4%) |  | 90 (62.9%) |  | 34 (53.1%) |  |
| **Age (years)** | 0 | 63 (48 – 77) | 0 | 64 (51 – 77) | 0 | 71 (55 – 80) | **0.048** |
| **Comorbidities** |  |  |  |  |  |  |  |
| Current smoker | 85 | 25 (6.6%) | 32 | 8 (5.6%) | 0 | 1 (1.6%) | 0.152 |
| Obesity (BMI > 25) | 69 | 155 (40.9%) | 0 | 69 (48.3%) | 0 | 36 (56.3%) | 0.561 |
| Diabetes type II | 1 | 78 (20.6%) | 0 | 26 (18.2%) | 0 | 15 (23.4%) | 0.667 |
| Hypertension | 1 | 188 (49.6%) | 15 | 65 (45.5%) | 0 | 32 (50.0%) | 0.979 |
| Cardiovascular disease  (any) | 2 | 103 (27.2%) | 21 | 33 (23.1%) | 0 | 15 (23.4%) | 0.809 |
| Coronary heart disease | 1 | 50 (13.2%) | 24 | 20 (14.0%) | 0 | 7 (10.9%) | 0.484 |
| Chronic heart failure | 1 | 30 (7.9%) | 1 | 4 (2.8%) | 0 | 3 (4.7%) | 0.087 |
| Atrial fibrillation | 1 | 53 (14.0%) | 0 | 12 (8.4%) | 0 | 11 (17.2%) | 0.133 |
| Peripheral arterial disease | 1 | 17 (4.5%) | 0 | 6 (4.2%) | 0 | 2 (3.1%) | 0.880 |
| Chronic obstructive  pulmonary disease | 1 | 34 (9.0%) | 22 | 9 (6.3%) | 0 | 4 (6.3%) | 0.703 |
| Asthma | 1 | 16 (4.2%) | 0 | 10 (7.0%) | 0 | 0 (0.0%) | 0.074 |
| **Hypo- / Hyperthyroidism** | 1 | 34 (9.0%) | 0 | 23 (16.1%) | 0 | 2 (3.1%) | **0.008** |
| **Chronic renal**  **Insufficiency** | 1 | 48 (12.7%) | 0 | 7 (4.9%) | 0 | 10 (15.6%) | **0.019** |
| **Chronic liver disease** | 2 | 14 (3.7%) | 0 | 13 (9.1%) | 0 | 2 (3.1%) | **0.032** |
| Malignancy | 1 | 32 (8.4%) | 0 | 20 (14.0%) | 0 | 7 (10.9%) | 0.170 |
| **Symptoms at admission** |  |  |  |  |  |  |  |
| **Asymptomatic** | 1 | 34 (9.0%) | 0 | 5 (3.5%) | 0 | 0 (0.0%) | **0.006** |
| **Fatigue** | 1 | 209 (55.1%) | 2 | 68 (47.6%) | 0 | 22 (34.4%) | **0.006** |
| **Cough** | 1 | 235 (62.0%) | 4 | 107 (74.8%) | 0 | 42 (65.6%) | **0.007** |
| Fever | 1 | 224 (59.1%) | 2 | 84 (58.7%) | 0 | 45 (70.3%) | 0.238 |
| **Requirement of oxygen** | 1 | 155 (40.9%) | 11 | 46 (32.2%) | 0 | 37 (57.8%) | **0.009** |
| Dyspnea | 1 | 151 (39.8%) | 8 | 63 (44.1%) | 0 | 19 (29.7%) | 0.071 |
| Diarrhea | 1 | 56 (14.8%) | 3 | 21 (14.7%) | 0 | 15 (23.4%) | 0.207 |
| Sore throat | 2 | 37 (9.8%) | 3 | 15 (10.5%) | 0 | 1 (1.6%) | 0.080 |
| **Nausea or vomiting** | 1 | 33 (8.7%) | 3 | 24 (16.8%) | 0 | 13 (20.3%) | **0.003** |
| **Vital Signs at admission** |  |  |  |  |  |  |  |
| Temperature (°C) | 67 | 36.7 (36.3 – 37.8) | 14 | 37.0 (36.3 – 37.8) | 64 | NA | 0.655 |
| Pulse rate  (beats per minute) | 30 | 85 (75 – 97) | 15 | 86 (75 – 97) | 64 | NA | 0.656 |
| Systolic arterial pressure (mmHg) | 25 | 130 (120 – 140) | 14 | 132 (119 – 145) | 64 | NA | 0.591 |
| Diastolic arterial pressure  (mmHg) | 27 | 80 (70 – 84) | 14 | 78 (70 – 85) | 64 | NA | 0.348 |
| Respiratory rate  (breaths per minute) | 81 | 20 (16 – 24) | 111 | 20 (16 – 24) | 64 | NA | 0.479 |
| SpO_2_ at ambient air (%) | 36 | 95 (93 – 97) | 13 | 95 (93 – 97) | 64 | NA | 0.560 |
| **COVID-19 classification at admission^†^** | 0 |  | 0 |  | 0 |  | **< 0.001** |
| Mild |  | 90 (23.7%) |  | 34 (23.8%) |  | 2 (3.1%) |  |
| Moderate |  | 166 (43.8%) |  | 55 (38.5%) |  | 19 (29.7%) |  |
| Severe |  | 99 (26.1%) |  | 38 (26.6%) |  | 28 (43.8%) |  |
| Critical |  | 24 (6.3%) |  | 16 (11.2%) |  | 15 (23.4%) |  |
| **Anticoagulation** |  |  |  |  |  |  |  |
| LMWH | 4 | 276 (72.8%) | 0 | 99 (69.2%) | 0 | 44 (68.8%) | 0.054 |
| **NOAC** | 0 | 53 (14.0%) | 0 | 2 (1.4%) | 0 | 7 (10.9%) | **< 0.001** |
| **Additional treatment** |  |  |  |  |  |  |  |
| **Corticosteroids** | **1** | **127 (33.6%)** | **0** | **35 (24.5%)** | **0** | **3 (4.7%)** | **< 0.001** |
| **Remdesivir/Favipiravir** | **0** | **37 (9.8%)** | **0** | **40 (28.0%)** | **0** | **0 (0.0%)** | **< 0.001** |
| **Lopinavir/Ritonavir** | **0** | **85 (22.4%)** | **0** | **0 (0.0%)** | **0** | **0 (0.0%)** | **< 0.001** |
| **Camostat** | **0** | **37 (9.8%)** | **0** | **0 (0.0%)** | **0** | **0 (0.0%)** | **< 0.001** |
| **Clinical Characteristics** |  |  |  |  |  |  |  |
| Total hospitalization (days) | 0 | 10 (6 – 16) | 0 | 10 (6 – 19) | 0 | 13 (5 – 29) | 0.099 |
| **Admission to ICU** | 0 | 57 (15.0%) | 0 | 41 (28.7%) | 0 | 13 (20.3%) | **0.002** |
| **Invasive ventilation** | 0 | 27 (7.1%) | 0 | 31 (21.7%) | 0 | 8 (12.5%) | **< 0.001** |
| Non-survivors | 0 | 58 (15.3%) | 0 | 17 (11.9%) | 0 | 13 (20.3%) | 0.283 |
| KFJ = Kaiser Franz Josef Hospital, MUI = Medical University Innsbruck, KUH-JKU = Kepler University Hospital and Johannes Kepler University, IQR = interquartile range, BMI = body mass index, NA = not assessed, mmHg = millimeter mercury, SpO_2_= peripheral capillary oxygen saturation, COVID-19 = Corona virus disease 2019, LMWH = low molecular weight heparin, NOAC = non-vitamin K anticoagulants, ICU = intensive care unit  **^†^** COVID-19 classification was performed according to the guidelines issued by the World Health Organization in mild (fever <38°C, no dyspnea, no pneumonia), moderate (fever, respiratory symptoms, pneumonia), severe (respiratory distress with respiratory rate ≥30 breaths per minute, SpO_2_ < 93% at rest) and critical (respiratory failure with requirement of mechanical ventilation, requirement of ICU) | | | | | | | |

| **Supplemental Table 3a. Missing Data** | | | | |
| --- | --- | --- | --- | --- |
|  | **Entire Cohort**  **(N=586)** | **Survivors**  **(N=498)** | **Non-Survivors**  **(N=88)** |  |
| **Parameter** | **N (%)** | **N (%)** | **N (%)** | **p-value** |
| Sex | 0 (0.0%) | 0 (0.0%) | 0 (0.0%) | NA |
| Age (years) | 0 (0.0%) | 0 (0.0%) | 0 (0.0%) | NA |
| Comorbidities |  |  |  |  |
| **Current smoker** | 117 (20.0%) | 89 (17.9%) | 28 (31.8%) | **0.003** |
| **Obesity (BMI > 25)** | 69 (11.8%) | 41 (8.2%) | 28 (31.8%) | **< 0.001** |
| Diabetes type II | 1 (0.2%) | 0 (0.0%) | 1 (1.1%) | 0.150* |
| Hypertension | 16 (2.7%) | 15 (3.0%) | 1 (1.1%) | 0.488* |
| Cardiovascular disease (any) | 23 (3.9%) | 19 (3.8%) | 4 (4.5%) | 0.765* |
| Coronary heart disease | 25 (4.2%) | 19 (3.8%) | 6 (6.8%) | 0.246* |
| Chronic heart failure | 2 (0.3%) | 1 (0.2%) | 1 (1.1%) | 0.278* |
| Atrial fibrillation | 1 (0.2%) | 0 (0.0%) | 1 (1.1%) | 0.150* |
| Peripheral arterial disease | 1 (0.2%) | 0 (0.0%) | 1 (1.1%) | 0.150* |
| Chronic obstructive pulmonary disease | 23 (3.9%) | 18 (3.6%) | 5 (5.7%) | 0.369* |
| Asthma | 1 (0.2%) | 0 (0.0%) | 1 (1.1%) | 0.150* |
| Hypo- / Hyperthyroidism | 1 (0.2%) | 0 (0.0%) | 1 (1.1%) | 0.150* |
| Chronic renal insufficiency | 1 (0.2%) | 0 (0.0%) | 1 (1.1%) | 0.150* |
| **Chronic liver disease** | 2 (0.3%) | 0 (0.0%) | 2 (2.3%) | **0.022*** |
| Malignancy | 1 (0.2%) | 0 (0.0%) | 1 (1.1%) | 0.150* |
| Symptoms at admission |  |  |  |  |
| Asymptomatic | 1 (0.2%) | 0 (0.0%) | 1 (1.1%) | 0.150* |
| Fatigue | 3 (0.5%) | 1 (0.2%) | 2 (2.3%) | 0.060* |
| **Cough** | 5 (0.9%) | 2 (0.4%) | 3 (3.4%) | **0.026*** |
| Fever | 3 (0.5%) | 1 (0.2%) | 2 (2.3%) | 0.060* |
| **Requirement of oxygen** | 12 (2.0%) | 7 (1.4%) | 5 (5.7%) | **0.023*** |
| **Dyspnea** | 9 (1.5%) | 5 (1.0%) | 4 (4.5%) | **0.033*** |
| Diarrhea | 4 (0.7%) | 2 (0.4%) | 2 (2.3%) | 0.109* |
| Sore throat | 5 (0.9%) | 3 (0.6%) | 2 (2.3%) | 0.164* |
| Nausea or vomiting | 4 (0.7%) | 2 (0.4%) | 2 (2.3%) | 0.109* |
| Vital Signs at admission |  |  |  |  |
| Temperature (°C) | 145 (24.7%) | 119 (23.9%) | 26 (29.5%) | 0.258 |
| **Pulse rate (beats per minute)** | 109 (18.6%) | 81 (16.3%) | 28 (31.8%) | **0.001** |
| **Systolic arterial pressure (mmHg)** | 103 (17.6%) | 80 (16.1%) | 23 (26.1%) | **0.022** |
| **Diastolic arterial pressure (mmHg)** | 105 (17.9%) | 82 (16.5%) | 23 (26.1%) | **0.029** |
| **Respiratory rate (breaths per minute)** | 256 (43.6%) | 209 (42.0%) | 47 (53.4%) | **0.046** |
| **SpO_2_ at ambient air (%)** | 113 (19.3%) | 89 (17.9%) | 24 (27.3%) | **0.039** |
| COVID-19 classification at admission^†^ | 0 (0.0%) | 0 (0.0%) | 0 (0.0%) | NA |
| Clinical Characteristics |  |  |  |  |
| Total hospitalization (days) | 0 (0.0%) | 0 (0.0%) | 0 (0.0%) | NA |
| Admission to ICU | 0 (0.0%) | 0 (0.0%) | 0 (0.0%) | NA |
| Invasive ventilation | 0 (0.0%) | 0 (0.0%) | 0 (0.0%) | NA |
| **Supplemental Table 3b. Missing Data** | | | | |
|  | **Entire Cohort**  **(N=586)** | **Survivors**  **(N=498)** | **Non-Survivors**  **(N=88)** |  |
| **Parameter** | **N (%)** | **N (%)** | **N (%)** | **p-value** |
| Hemoglobin (g/dL) | 173 (29.5%) | 147 (29.5%) | 26 (29.5%) | 0.996 |
| Red blood cell count (x10^12^/L) | 139 (23.7%) | 118 (23.7%) | 21 (23.9%) | 0.973 |
| Platelet count (x10^9^/L) | 159 (27.1%) | 137 (27.5%) | 22 (25.0%) | 0.625 |
| Leukocyte count (x10^9^/L) | 159 (27.1%) | 137 (27.5%) | 22 (25.0%) | 0.625 |
| Lymphocyte count (x10^9^/L) | 144 (24.6%) | 124 (24.9%) | 20 (22.7%) | 0.663 |
| C-reactive protein (mg/L) | 162 (27.6%) | 139 (27.9%) | 23 (26.1%) | 0.731 |
| D-dimer (mg/L) | 366 (62.5%) | 308 (61.8%) | 58 (65.9%) | 0.468 |
| Prothrombin time (%) | 229 (39.1%) | 189 (38.0%) | 40 (45.5%) | 0.184 |
| International normalized ratio | 274 (46.8%) | 229 (46.0%) | 45 (51.1%) | 0.372 |
| Activated partial thromboplastin time (s) | 256 (43.7%) | 215 (43.2%) | 41 (46.6%) | 0.551 |
| IQR = interquartile range, BMI = body mass index, mmHg = millimeter mercury, SpO_2_= peripheral capillary oxygen saturation, COVID-19 = Corona virus disease 2019, ICU = intensive care unit.  **^†^** COVID-19 classification was performed according to the guidelines issued by the World Health Organization in mild (fever <38°C, no dyspnea, no pneumonia), moderate (fever, respiratory symptoms, pneumonia), severe (respiratory distress with respiratory rate ≥30 breaths per minute, SpO_2_ < 93% at rest) and critical (respiratory failure with requirement of mechanical ventilation, requirement of ICU)  * Fisher’s exact test | | | | |

| **Supplemental Table 4. Cox-Regression Analysis with Selected Confounders in Multiple Imputation Dataset** | | | | | | |
| --- | --- | --- | --- | --- | --- | --- |
|  | **Univariable Cox-regression** | | | **Multivariable Cox-regression** | | |
| **Parameter** | **HR** | **95%CI** | **p-value** | **HR** | **95%CI** | **p-value** |
| **Age (years)** | **1.075** | **1.055 – 1.096** | **< 0.001** | **1.086** | **1.057 – 1.115** | **< 0.001** |
| Comorbidities |  |  |  |  |  |  |
| Diabetes type II | 1.317 | 0.835 – 2.077 | 0.237 | 1.064 | 0.653 – 1.733 | 0.803 |
| Cardiovascular disease (any) | 3.493 | 2.248 – 5.427 | < 0.001 | 1.502 | 0.856 – 2.633 | 0.156 |
| Chronic obstructive pulmonary disease | 1.682 | 0.935 – 3.024 | 0.082 | 0.745 | 0.393 – 1.413 | 0.367 |
| **Chronic renal insufficiency** | **3.466** | **2.205 – 5.448** | **< 0.001** | **1.730** | **1.035 – 2.893** | **0.037** |
| **Malignancy** | **1.978** | **1.163 – 3.364** | **0.012** | **2.395** | **1.338 – 4.286** | **0.003** |
| Symptoms at admission |  |  |  |  |  |  |
| **Requirement of oxygen** | **2.077** | **1.306 – 3.304** | **0.002** | **1.710** | **1.036 – 2.823** | **0.036** |
| COVID-19 classification  at admission^†^ | 1.654 | 1.303 – 2.099 | < 0.001 | 1.307 | 0.978 – 1.746 | 0.071 |
| Clinical characteristics |  |  |  |  |  |  |
| Admission to ICU | 1.161 | 0.738 – 1.827 | 0.517 | 1.673 | 0.733 – 3.822 | 0.222 |
| Invasive ventilation | 1.196 | 0.722 – 1.982 | 0.487 | 1.839 | 0.784 – 4.313 | 0.161 |
| Anticoagulation |  |  |  |  |  |  |
| **LMWH** | **0.413** | **0.270 – 0.634** | **< 0.001** | **0.539** | **0.336 – 0.866** | **0.011** |
| Additional treatment |  |  |  |  |  |  |
| Remdesivir/Favipiravir | 1.082 | 0.648 – 1.805 | 0.764 | 1.153 | 0.605 – 2.195 | 0.665 |
| HR = hazard ratio, 95%CI = 95% confidence interval, ICU = intensive care unit, LMWH = low molecular weight heparin.  **^†^** COVID-19 classification was performed according to the guidelines issued by the World Health Organization in mild (fever <38°C, no dyspnea, no pneumonia), moderate (fever, respiratory symptoms, pneumonia), severe (respiratory distress with respiratory rate ≥30 breaths per minute, SpO_2_ < 93% at rest) and critical (respiratory failure with requirement of mechanical ventilation, requirement of ICU). | | | | | | |

| **Supplemental Table 5. Patient demographics according type of anticoagulation*** | | | | | | | |  |
| --- | --- | --- | --- | --- | --- | --- | --- | --- |
|  | **LMWH**  **(N=419)** | **NOAC**  **(N=62)** | **no LMWH /**  **no NOAC**  **(N=101)** | **Statistical comparison** | | | | |
| **Parameter** | **N (%)**  **Median (IQR)** | **N (%)**  **Median (IQR)** | **N (%)**  **Median (IQR)** | **p-value_1_** | **p-value_2_** | **p-value_3_** | **p-value_4_** | |
| **Sex** |  |  |  | 0.604 | 0.867 | 0.341 | 0.422 | |
| Female | 171 (40.8%) | 26 (41.9%) | 36 (35.6%) |  |  |  |  | |
| Male | 248 (59.2%) | 36 (58.1%) | 65 (64.4%) |  |  |  |  | |
| **Age (years)** | 62 (48 – 77) | 74 (63 – 81) | 66 (50 – 76) | **< 0.001** | **< 0.001** | 0.316 | **0.003** | |
| **Comorbidities** |  |  |  |  |  |  |  | |
| Current smoker | 25 (6.0%) | 2 (3.2%) | 7 (6.9%) | 0.588 | 0.417 | 0.605 | 0.300 | |
| Obesity (BMI > 25) | 193 (46.1%) | 27 (43.5%) | 39 (38.6%) | 0.406 | 0.498 | 0.295 | 0.204 | |
| Diabetes type II | 82 (19.6%) | 17 (27.4%) | 18 (17.8%) | 0.299 | 0.154 | 0.721 | 0.157 | |
| Hypertension | 200 (47.7%) | 38 (61.3%) | 45 (44.6%) | 0.163 | 0.069 | 0.788 | 0.088 | |
| **Cardiovascular disease**  **(any)** | 82 (19.6%) | 37 (59.7%) | 31 (30.7%) | **< 0.001** | **< 0.001** | **0.004** | **0.002** | |
| **Coronary heart disease** | 45 (10.7%) | 14 (22.6%) | 17 (16.8%) | **0.013** | **0.011** | **0.039** | 0.603 | |
| **Chronic heart failure** | 21 (5.0%) | 9 (14.5%) | 7 (6.9%) | **0.016** | **0.004** | 0.415 | 0.124 | |
| **Atrial fibrillation** | 28 (6.7%) | 29 (46.8%) | 18 (17.8%) | **< 0.001** | **< 0.001** | **< 0.001** | **< 0.001** | |
| Peripheral arterial disease | 16 (3.8%) | 5 (8.1%) | 4 (4.0%) | 0.302 | 0.127 | 0.933 | 0.272 | |
| **Chronic obstructive**  **pulmonary disease** | 23 (5.5%) | 13 (21.0%) | 11 (10.9%) | **< 0.001** | **< 0.001** | **0.025** | 0.146 | |
| Asthma | 21 (5.0%) | 1 (1.6%) | 4 (4.0%) | 0.467 | 0.232 | 0.671 | 0.393 | |
| Hypo- / Hyperthyroidism | 39 (9.3%) | 7 (11.3%) | 13 (12.9%) | 0.521 | 0.620 | 0.269 | 0.748 | |
| **Chronic renal insufficiency** | 31 (7.4%) | 18 (29.0%) | 16 (15.8%) | **< 0.001** | **< 0.001** | **0.007** | **0.048** | |
| Chronic liver disease | 22 (5.3%) | 3 (4.8%) | 4 (4.0%) | 0.872 | 0.888 | 0.603 | 0.799 | |
| Malignancy | 37 (8.8%) | 10 (16.1%) | 11 (10.9%) | 0.188 | 0.071 | 0.501 | 0.345 | |
| **Symptoms at admission** |  |  |  |  |  |  |  | |
| Asymptomatic | 23 (5.5%) | 6 (9.7%) | 9 (8.9%) | 0.264 | 0.198 | 0.201 | 0.869 | |
| **Fatigue** | 226 (53.9%) | 33 (53.2%) | 39 (38.6%) | **0.023** | 0.886 | **0.006** | 0.077 | |
| Cough | 279 (66.6%) | 35 (56.5%) | 67 (66.3%) | 0.240 | 0.100 | 0.908 | 0.150 | |
| Fever | 261 (62.3%) | 32 (51.6%) | 57 (56.4%) | 0.190 | 0.098 | 0.302 | 0.503 | |
| Requirement of oxygen | 170 (40.6%) | 33 (53.2%) | 32 (31.7%) | 0.078 | 0.064 | 0.322 | **0.027** | |
| Dyspnea | 170 (40.6%) | 26 (41.9%) | 35 (34.7%) | 0.591 | 0.908 | 0.323 | 0.430 | |
| Diarrhea | 65 (15.5%) | 13 (21.0%) | 13 (12.9%) | 0.397 | 0.288 | 0.511 | 0.179 | |
| Sore throat | 44 (10.5%) | 2 (3.2%) | 7 (6.9%) | 0.122 | 0.066 | 0.279 | 0.308 | |
| Nausea or vomiting | 58 (13.8%) | 5 (8.1%) | 7 (6.9%) | 0.094 | 0.202 | 0.060 | 0.801 | |
| **Vital Signs at admission** |  |  |  |  |  |  |  | |
| **Temperature (°C)** | 36.9 (36.4 – 37.8) | 36.5 (36.2 – 37.5) | 36.5 (36.1 – 37.9) | **0.046** | 0.052 | 0.068 | 0.875 | |
| Pulse rate (beats per minute) | 86 (75 – 97) | 81 (72 – 93) | 84 (75 – 100) | 0.459 | 0.216 | 0.949 | 0.314 | |
| Systolic arterial pressure  (mmHg) | 130 (120 – 142) | 130 (110 – 140) | 131 (120 – 142) | 0.483 | 0.368 | 0.506 | 0.226 | |
| Diastolic arterial pressure  (mmHg) | 80 (70 – 84) | 80 (70 – 86) | 80 (71 – 85) | 0.648 | 0.768 | 0.401 | 0.433 | |
| Respiratory rate  (breaths per minute) | 20 (16 – 24) | 18 (16 – 22) | 18 (16 – 24) | 0.673 | 0.378 | 0.853 | 0.565 | |
| SpO_2_ at ambient air (%) | 95 (93 – 97) | 95 (93 – 96) | 95 (93 – 97) | 0.326 | 0.127 | 0.797 | 0.364 | |
| **COVID-19 classification**  **at admission^†^** |  |  |  | 0.481 | 0.784 | 0.159 | 0.915 | |
| Mild | 91 (21.7%) | 13 (21.0%) | 21 (20.8%) |  |  |  |  | |
| Moderate | 177 (42.2%) | 24 (38.7%) | 39 (38.6%) |  |  |  |  | |
| Severe | 119 (28.4%) | 18 (29.0%) | 26 (25.7%) |  |  |  |  | |
| Critical | 32 (7.6%) | 7 (11.3%) | 15 (14.9%) |  |  |  |  | |
| **Additional treatments** |  |  |  |  |  |  |  | |
| **Corticosteroids** | **123 (29.4%)** | **24 (38.7%)** | **16 (15.8%)** | **0.003** | 0.139 | **0.006** | **0.001** | |
| Remdesivir/Favipiravir | 56 (13.4%) | 5 (8.1%) | 15 (14.9%) | 0.431 | 0.242 | 0.696 | 0.200 | |
| **Lopinavir/Ritonavir** | **50 (11.9%)** | **15 (24.2%)** | **17 (16.8%)** | **0.024** | **0.008** | 0.187 | 0.251 | |
| **Camostat** | **27 (6.4%)** | **10 (16.1%)** | **0 (0.0%)** | **< 0.001** | **0.008** | **0.009** | **< 0.001** | |
| **Clinical characteristics** |  |  |  |  |  |  |  | |
| **Total hospitalization (days)** | 10 (6 – 16) | 13 (9 – 21) | 8 (4 – 23) | **0.020** | **0.006** | 0.590 | **0.025** | |
| **Admission to ICU** | 68 (16.2%) | 11 (17.7%) | 30 (29.7%) | **0.008** | 0.764 | **0.002** | 0.088 | |
| **Invasive ventilation** | 38 (9.1%) | 3 (4.8%) | 23 (22.8%) | **< 0.001** | 0.266 | **< 0.001** | **0.002** | |
| **Non-survivors** | 44 (10.5%) | 9 (14.5%) | 33 (32.7%) | **< 0.001** | 0.346 | **< 0.001** | **0.010** | |
| * Information on use of anticoagulation is missing in 4 patients.  p-value_1_ = Comparison of distribution over all three sub-groups  p-value_2_ = Comparison of distribution between LMWH and NOAC sub-groups.  p-value_3_ = Comparison of distribution between LMWH and no LMWH / no NOAC sub-groups.  p-value_4_ = Comparison of distribution between NOAC and no LMWH / no NOAC sub-groups.  LMWH = low molecular weight heparin, NOAC = non-vitamin K anticoagulants, IQR = interquartile range, BMI = body mass index, mmHg = millimeter mercury, SpO_2_= peripheral capillary oxygen saturation, COVID-19 = Corona virus disease 2019, ICU = intensive care unit  **^†^** COVID-19 classification was performed according to the guidelines issued by the World Health Organization in mild (fever <38°C, no dyspnea, no pneumonia), moderate (fever, respiratory symptoms, pneumonia), severe (respiratory distress with respiratory rate ≥30 breaths per minute, SpO_2_ < 93% at rest) and critical (respiratory failure with requirement of mechanical ventilation, requirement of ICU) | | | | | | | |  |

**Supplemental Methods**

**Plasma preparation and evaluation of markers of hemostasis and NET formation**

106 patients were included after written informed consent within the first week of hospitalization and blood was taken every second day (or third day in case of a weekend in between) for up to four blood withdrawals. Blood was drawn into citrate, theophylline, adenosine and dipyridamole (CTAD) and plasma was prepared as previously described^1^. After the last centrifugation step, plasma was stored at -80°C until further processing, ensuring a single freeze/thaw cycle.

Relevant hemostatic biomarkers, including fibrinogen, antithrombin, plasminogen and prothrombin were evaluated from patient plasma using a commercially available multiplex assay (LEGENDPlex™ Multi-Analyte Flow Assay Kit Human Fibrinolysis Panel, BioLegend®, San Diego, USA). The beads-based assay was carried out according to manufacturers’ instructions and fluorescence was measured using a CytoflexS flow cytometer and CytExpert 2.3 software (Beckman Coulter, Vienna, Austria). Absolute values were calculated from beads-based standards after importing flow cytometry data into LEGENDplex™ data analysis software v8.0 (BioLegend®).

In order to evaluate NET formation in circulation, complexes of citrullinated histone 3 and DNA (H3Cit-DNA) were assessed according to Thalin. et al.^2^ using semi-synthetic human designer nucleosomes (EpiCypher®) as assay standard. In addition, cell-free DNA (cfDNA) in plasma was quantified using a Quant-iT™ PicoGreen®dsDNA Assay Kit (Thermo Fisher Scientiﬁc, Waltham, MA, USA) according to manufacturers’ instructions^3^. Of note, cfDNA is often associated to NET formation, while being a general marker for cell death in circulation.

**Supplemental Statistical Analysis**

Cohort comparison and comparison of predictive markers for in-hospital mortality

Collected data on patient demographics and laboratory parameters at admission were compared between survivors and non-survivors using Mann-Whitney-U tests, Chi-square or Fisher’s exact tests.

To test whether the potential predictors age, D-Dimer, PT, INR and aPTT indeed predict the binary outcome death or survival irrespective of when the event took place, they were each entered in separate univariable logistic regression models. In order to evaluate, whether addition of any hematologic biomarker to age improves the prognostic value, multivariable logistic regression was fit. The resulting predicted death probabilities for each patient were plotted against the actual outcome to visualize how well the potential predictor of death in fact discriminates between death and survival. The given p-value refers to the hypothesis that Exp(B), i.e. the odds ratio is not equal to one. The prognostic value of age alone and age with addition of each one hematologic biomarker was compared using receiver operating characteristic (ROC) analysis.

Missing values and multiple imputation

Missing values in all evaluated variables of patient demographics including laboratory data were evaluated. Proportions of missing values were statistically compared between survivors and non-survivors using Mann-Whitney-U tests, Chi-square or Fisher’s exact tests. In order to account for missing values relevant for comparison of patient demographics and for prediction of in-hospital mortality, logistic regression based multiple imputation was conducted. Data on symptoms at admission (asymptomatic, fatigue, cough, fever, requirement of oxygen, dyspnea, diarrhea, sore throat, nausea/vomiting), co-morbidities (smoking status, obesity, diabetes type II, hypertension, cardiovascular diseases, chronic obstructive pulmonary disease, asthma, hypo-/hyperparathyroidism, chronic renal insufficiency, chronic liver disease, malignancy), vital signs at admission (temperature, pulse rate, systolic/diastolic arterial pressure, respiratory rate, oxygen saturation at ambient air), and laboratory data (hemoglobin, red blood cell count, platelet count, leukocyte count, lymphocyte count, C-reactive protein, D-dimer, prothrombin time, international normalized ratio, activated partial thromboplastin time) were imputed in a total of 20 data sets. While nominal variables (i.e. symptoms at admission and co-morbidities) were imputed dichotomously (yes/no), metric parameters were subjected to secondary conditions. In particular, information on vital signs were constrained between the observed minimum and maximum values in the original data set allowing for minor deviations (i.e. temperature 34.0 °C ≤ x ≤ 42 °C, pulse rate 40 bpm ≤ x ≤ 130 bpm, systolic arterial pressure 80 mmHg ≤ x ≤ 200 mmHg, diastolic arterial pressure 40 mmHg ≤ x ≤ 140 mmHg, respiratory rate 10 bpm ≤ x ≤ 100 bpm, oxygen saturation at ambient air 70 % ≤ x ≤ 100%). Laboratory values were imputed with the only limitation being x > 0 in case for continuously available routine laboratory measurement (hemoglobin, red blood cell count, platelet count, leukocyte count, lymphocyte count, C-reactive protein). For laboratory tests with an upper limit this limit was used as an upper cut-off for imputed values (i.e. D-dimer 0 < x ≤ 35.2 mg/dL, prothrombin time 0 < x ≤ 140%, international normalized ratio 0 < x ≤ 7, activated prothrombin time 0 < x ≤ 160 seconds). Of note, values were only imputed for missing data at admission, while evaluated parameters throughout the entire hospitalization served as predictors for multiple imputation, meaning that laboratory values on any day during hospitalization allowed better estimation of imputed values. Further, age, sex, COVID-19 classification, and time point (for laboratory values) served as predictors for imputed values in addition to the variables being imputed.

Statistical analysis of differences in baseline characteristics and routine laboratory parameters between survivors and non-survivors was further evaluated in the imputed data sets (n=20). In order to allow for unbiased interpretation, the p-values estimated in each data set were visualized and given in Supplemental Figure 1. Additionally, logistic regression for prediction of in-hospital mortality was performed using the imputed data sets. Here, odds ratios, 95% confidence intervals and p-values are given for the combined set of all imputed data sets.

Of note, data on use of anticoagulation and outcome were not imputed due to the minor percentage of missing values (<1%). Thus, evaluations including these variables were only performed in the original data set.

Mixed linear models

The time courses of D-dimer levels were explored using a mixed model approach. As data were heavily right-skewed, they were log-transformed before analysis. To test the main hypothesis regarding D-dimer time courses, the disease group with the three levels ‘uncomplicated’, ‘ICU’ and ‘non-survivor’ was specified as fixed factor, and the time since admission in days was used as fixed continuous predictor. The interaction term between these two variables was used to answer the primary research question whether the slopes differ between groups in general. To further show which of the three groups differ in general regarding slope in the sense of a post hoc test, the p-value corresponding to the estimates of the three fixed interaction terms a) uncomplicated×time b) ICU×time and c) death×time were taken and corrected for multiplicity according to Bonferroni-Holm, i.e. the lowest p-value was multiplied by 3, the second-lowest by two and the highest p-value was left unchanged. Before testing hypotheses based on the fixed effects, the model had to account for the dependency of values taken from the same subjects. At first, a random factor was applied, allowing for an individual intercept for each patient. Next a random slope was introduced by adding a random predictor ‘time’, which led to a smaller Akaike information criterion. However, as some individual curves are bent over time, a random time×time interaction was introduced to allow individually curved time courses of log D-dimer. Since this further reduced the AIC and led to a nicely normal distributed residual distribution, the model with the three random effects was used. To visualize the estimated means over time in each group the following syntax was used: “/EMMEANS=TABLES(Outcome) WITH(Day_Labor=x)”, whereby x was replaced by a day in steps of 0.5. The resulting least square means with 95% confidence intervals were plotted over the original data to allow the reader to decide how well the model fits the original data. An additional question was whether the observed differences in log D-dimer values were affected by whether LMWH or NOAC were taken or not. For this purpose, an additional binary fixed factor was introduced in a full factorial manner, i.e. including all interaction terms. The three-way interaction would answer the question if the slope differences between groups differ between patients taking LMWH, NOAC or not. The LMWH×time and NOAC×time interaction would mean that the slopes are generally different between patients taking LMHW or NOAC and those who did not, and the LMWH×group and NOAC×group interaction tested whether log D-dimer levels were differently affected by LMWH or NOAC in each group. Finally, the main effect of LMWH or NOAC tested whether mean log D-dimer levels (i.e. the geometric mean D-dimer levels) generally differed between patients taking LMWH or NOAC and those who did not, independent on group or time. As for LMWH and NOAC, differences in D-dimer dynamics were assessed between patients with mild to moderate and patients with severe to critical COVID-19 disease severity at admission.

In order to evaluate dynamics of NET markers in circulation an additional mixed model approach was fit. Here, circulating concentrations of H3Cit-DNA complexes and cfDNA entered the model as dependent variables. Further analysis was performed in parallel to the mixed model generated for D-dimer dynamics and depicted in the paragraph above.

Cox-regression analysis for in-hospital survival

To test whether the survival probability over time is different between patients taking LMWH or NOAC at admission and those who did not, a Cox proportional hazard regression approach was used. Initially, we observed a decrease in risk for mortality throughout the study period in parallel to an increase in the probability of receiving LMWH treatment. Consequently, the effect of time point of inclusion (i.e. the day of the study period on which the patient was admitted to hospital starting with the first patient on Feburary 14^th^ 2020), use of LMWH, and an interaction term time point of inclusion x LMWH were evaluated in a multivariable Cox-regression analysis for in-hospital survival. Importantly, the interaction term did not show a significant association with survival, while both time point of inclusion and LMWH use were significantly associated with survival in the consequently computed Cox-regression analysis excluding the interaction term. Thus, we observed that use of LMWH was associated with in-hospital survival independently from time of inclusion, while also time point of inclusion had an independent effect on patient survival. Next, age was entered in the model in addition to the main factor of interest, LMWH or NOAC use, as survival of COVID-19 patients heavily depends on age. The given P-value refers to the hypothesis whether Exp(B) is different from one while potential confounders are included in the model. Estimated survival curves for each group were plotted, and serious violations of the proportional hazard assumption was checked visually using partial Schoenfeld residuals for each variable separately. In additional analyses, confounders of LMWH use (i.e. presence of cardiovascular disease, chronic kidney failure) and additional treatments for COVID-19 (corticosteroids, remdesivir/favipiravir, lopinavir/ritonavir, camostat) entered the Cox-regression analysis in order to evaluate potential interaction with the association of LMWH use and improved hospital survival. Analogous analyses were performed with the event of a negative SARS-CoV-2 qPCR test.

All statistical analyses were performed with IBM SPSS statistics 26, graphs were generated with IBM SPSS statistics 26 and GraphPad Prism 8.4. Due to the exploratory character of all analyses, no adjustment for multiple testing was performed, results have to be interpreted accordingly. Only two-sided tests were used, and P-values ≤ 0.05 were considered statistically significant.

**References**

1. Mussbacher M, Schrottmaier WC, Salzmann M, Brostjan C, Schmid JA, Starlinger P, Assinger A. Optimized plasma preparation is essential to monitor platelet-stored molecules in humans. *PloS one* 2017;**12**:e0188921.

2. Thålin C, Aguilera K, Hall NW, Marunde MR, Burg JM, Rosell A, Daleskog M, Månsson M, Hisada Y, Meiners MJ, Sun ZW, Whelihan MF, Cheek MA, Howard SA, Saxena-Beem S, Noubouossie DF, Key NS, Sheikh SZ, Keogh MC, Cowles MW, Lundström S, Mackman N, Wallén H, Johnstone AL. Quantification of citrullinated histones: Development of an improved assay to reliably quantify nucleosomal H3Cit in human plasma. *Journal of thrombosis and haemostasis : JTH* 2020.

3. Mauracher LM, Posch F, Martinod K, Grilz E, Däullary T, Hell L, Brostjan C, Zielinski C, Ay C, Wagner DD, Pabinger I, Thaler J. Citrullinated histone H3, a biomarker of neutrophil extracellular trap formation, predicts the risk of venous thromboembolism in cancer patients. *Journal of thrombosis and haemostasis : JTH* 2018;**16**:508-518.
